# Double-edged role of mechanical stimuli and underlying mechanisms in cartilage tissue engineering

**DOI:** 10.3389/fbioe.2023.1271762

**Published:** 2023-11-20

**Authors:** Yao Jia, Hanxiang Le, Xianggang Wang, Jiaxin Zhang, Yan Liu, Jiacheng Ding, Changjun Zheng, Fei Chang

**Affiliations:** ^1^ Department of Orthopedics, The Second Hospital of Jilin University, Jilin, China; ^2^ The Second Bethune Clinical Medical College of Jilin University, Jilin, China; ^3^ The Fourth Treatment Area of Trauma Hip Joint Surgery Department, Tianjin Hospital, Tianjin, China

**Keywords:** mechanical stimuli, cartilage tissue engineering, mechanoreceptor, downstream pathway, biomaterials

## Abstract

Mechanical stimuli regulate the chondrogenic differentiation of mesenchymal stem cells and the homeostasis of chondrocytes, thus affecting implant success in cartilage tissue engineering. The mechanical microenvironment plays fundamental roles in the maturation and maintenance of natural articular cartilage, and the progression of osteoarthritis Hence, cartilage tissue engineering attempts to mimic this environment *in vivo* to obtain implants that enable a superior regeneration process. However, the specific type of mechanical loading, its optimal regime, and the underlying molecular mechanisms are still under investigation. First, this review delineates the composition and structure of articular cartilage, indicating that the morphology of chondrocytes and components of the extracellular matrix differ from each other to resist forces in three top-to-bottom overlapping zones. Moreover, results from research experiments and clinical trials focusing on the effect of compression, fluid shear stress, hydrostatic pressure, and osmotic pressure are presented and critically evaluated. As a key direction, the latest advances in mechanisms involved in the transduction of external mechanical signals into biological signals are discussed. These mechanical signals are sensed by receptors in the cell membrane, such as primary cilia, integrins, and ion channels, which next activate downstream pathways. Finally, biomaterials with various modifications to mimic the mechanical properties of natural cartilage and the self-designed bioreactors for experiment *in vitro* are outlined. An improved understanding of biomechanically driven cartilage tissue engineering and the underlying mechanisms is expected to lead to efficient articular cartilage repair for cartilage degeneration and disease.

## 1 Introduction

Articular cartilage is a hyaline cartilage tissue consisting of chondrocytes and a rich extracellular matrix (ECM), mainly composed of proteoglycans, collagen type II, and water (Uzieliene et al., 2021). It serves as a shock absorber and covers the joint surface to create a low friction and load-bearing environment for joint motion (Bernhard and Vunjak-Novakovic, 2016). The articular cartilage is incredibly sensitive to mechanical stimuli. Articular cartilage’s mechanical characteristics are influenced by the microstructure of ECM, which is defined mainly by the mechanical environment. Three overlapping zones that extend from the surface to the subchondral bone can be found in the adult articular cartilage (Hodgkinson et al., 2022). Within these zones, the morphology of chondrocytes, as well as the content and architecture of ECM, reflect the forces experienced during joint motion. For example, type II collagen fibers are oriented perpendicular to the joint surface to resist compressive loads and parallel to the joint surface to disperse shear pressures (Wang Z. H. et al., 2022).

The development, pathology, and regeneration of articular cartilage tissue are fundamentally influenced by biomechanics. Atrophy and acinetatropbia are frequently caused by super-reduced biomechanical loading, although irreparable damage can sometimes be caused by mechanical overload (Le et al., 2020). A 2022 study showed that excessive mechanical load affects the progression of OA by regulating cartilage degradation. Mouse and human cartilage experiments both revealed that high-strain mechanical stress induces GPX4-associated ferroptosis in chondrocytes from OA patients. Piezo1-mediated calcium ion inflow plays a major role in this process, which can be blocked by GsMTx4, an inhibitor of Piezo1 (Wang S. et al., 2022). Additionally, the chondrocytes’ physical microenvironment significantly affects the homeostasis and functionality of cartilage. Our previous research showed that biomechanical stimuli can improve cartilage regeneration. Bone marrow-derived cells extracted from patients during orthopedic surgery were divided into two groups, cultured in a rotating wall vessel (RWV) bioreactor or pellet culture as controls. The mechanical stimuli created by RWV significantly promote the formation of hyaline cartilage chondrogenic medium without scaffold. The content of glycosaminoglycan in the experimental group was significantly higher than that in the control group. This study suggests that stress stimulation may promote cells to form 3D structures autonomously, which can be used to construct scaffold-free 3D tissue-engineered cartilage *in vitro* (Sakai et al., 2009). In addition, our *in vivo* experiment demonstrated that appropriate mechanical stimulation can promote cartilage regeneration by enhancing the production of type II collagen. In the experimental group, gradual weight bearing was exerted 6 weeks after a full-thickness defect. The staining area of type II collagen antibody in the experimental group was significantly higher than that in the control group (Nishino et al., 2010b). In another experiment, we explored the long-term effects of mechanical stimuli on cartilage repair after a full-thickness defect. We first applied a hinged external fixation device in rabbits for 6 months. Then, we removed the device and let the rabbits roam free for 6 months. This technique promoted cartilage repair in the long term by increasing the content of type II collagen (Nishino et al., 2010a).

Incorporating the use of mechanical stimuli can fortify neotissue to impart the properties of actual articular cartilage. Through the development of bioreactors, the effect of various complex mechanical stimuli in MSCs, including compression, hydrostatic pressure, fluid shear stress, and osmotic pressure, can be achieved. Moreover, the content and architecture of the ECM structure might be recreated using a variety of modified biomaterials, to mimic the natural articular cartilage as intended for a mechanically competent replacement (Armiento et al., 2018).

**FIGURE 1:**
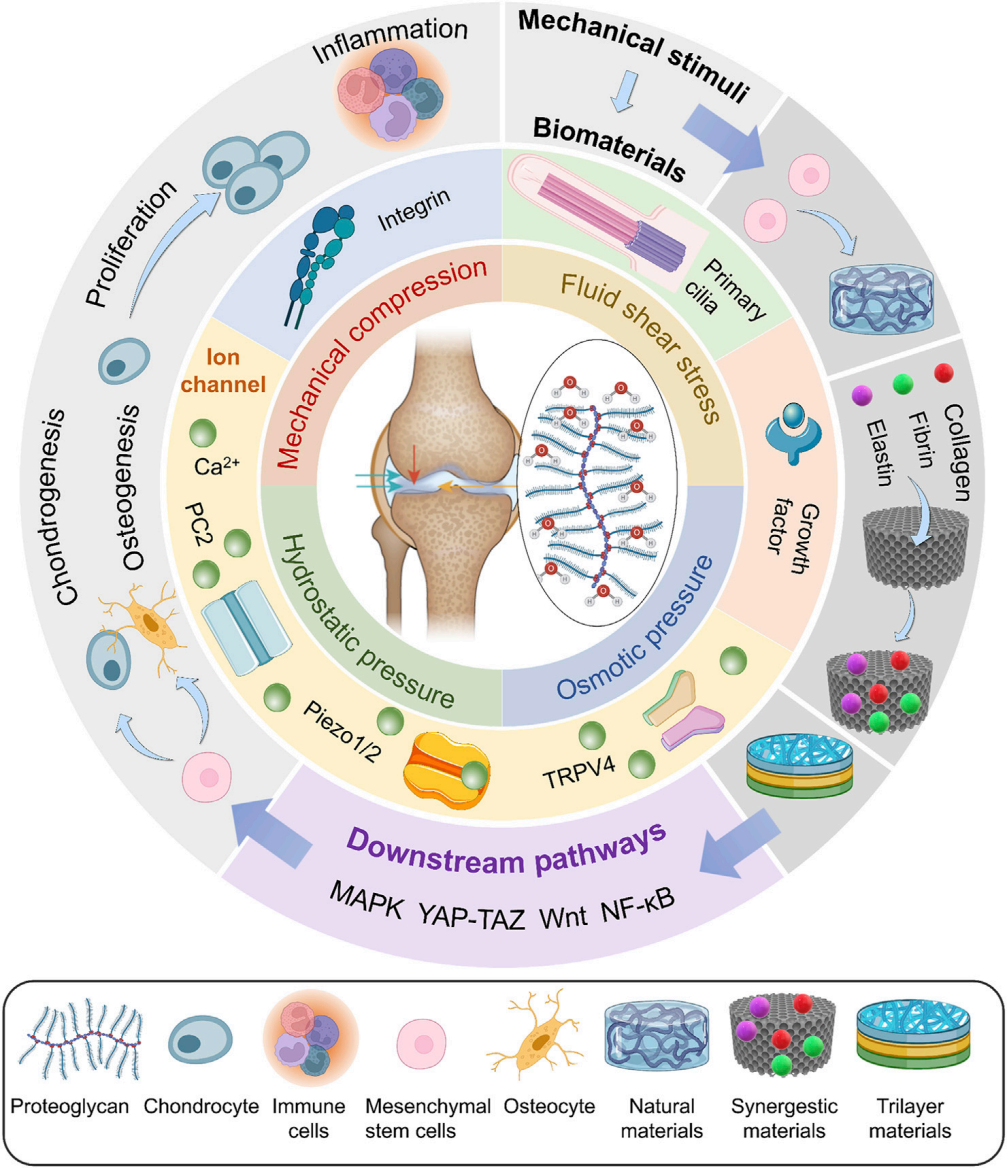
Bioreactors exert mechanical stimuli, including mechanical compression, fluid shear stress, osmotic pressure, and hydrostatic pressure, on various biomaterials. These signals are sensed by mechanosensors and regulate the differentiation of MSCs, proliferation of chondrocytes, and inflammation through downstream pathways.

Increasing attention is paid to the effect of mechanical stimuli on articular cartilage. However, the precise methods by which mechanical stimulation causes changes in chondrocytes as well as the proper magnitude have not yet been fully understood. To this end, this article provides an overview of the effect of several mechanical stimuli and these processes at the molecular level. A summary of the most well-understood biomaterials is also presented. By doing so, we provide a timely answer to the questions of what the effects of mechanical stimuli on articular cartilage are, which proteins play a crucial role in regulating this process, and how they transduce the mechanical stimuli into signals to the cell (Figure 1).

## 2 The composition and structure of articular cartilage

The articular surfaces of the bone are covered in hyaline cartilage, which provides a low-friction surface. Articular cartilage works to absorb and distribute stress in the mechanically demanding environment of the joint in addition to providing a low-friction surface (Fahy et al., 2018). These exceptional functional qualities are a result of the articular cartilage’s highly specialized composition and structure.

Molecular layers at the sliding cartilage surfaces reduce friction. Three main molecular species are implicated in forming such boundary layers: hyaluronic acid (HA), lubricin, and phosphatidylcholine liposomes (LPs), which, at the high physiological pressures (of order 100 atm), typical of the major joints, appear to produce reduced friction (values down to 10–3) (Lin and Klein, 2021).

Chondrocytes integrated in a rich extracellular matrix (ECM) make up the highly specialized tissue known as articular cartilage (Choi et al., 2018). Two phases can be distinguished within the articular cartilage from a material perspective. First, a framework of collagen fibers that primarily consists of type II, type IX, and type XI collagen is a solid phase that gives the tissue its general structure. Glycosaminoglycans (GAGs), proteoglycans, and glycoproteins make up a small portion of this network. The second phase is a liquid made up of water and electrolytes (Ca^2+^, K^+^, Na^+^, and Cl^−^), which contains all of the solid components. The ECM is made up of these two stages (Armiento et al., 2018). While collagen fibers provide tensile strength, the tissue is resistant to large compressive pressures due to the significant attraction of water to the negatively charged proteoglycans (Hua and Jiang, 2021). The porous matrix prevents interstitial fluid from escaping during mechanical compression, providing internal pressure to withstand the imposed load and protect the cells within the solid matrix (Schätti et al., 2015). The composition and structure of ECM determine its physical properties, which are highly specialized for weight bearing.

**FIGURE 2:**
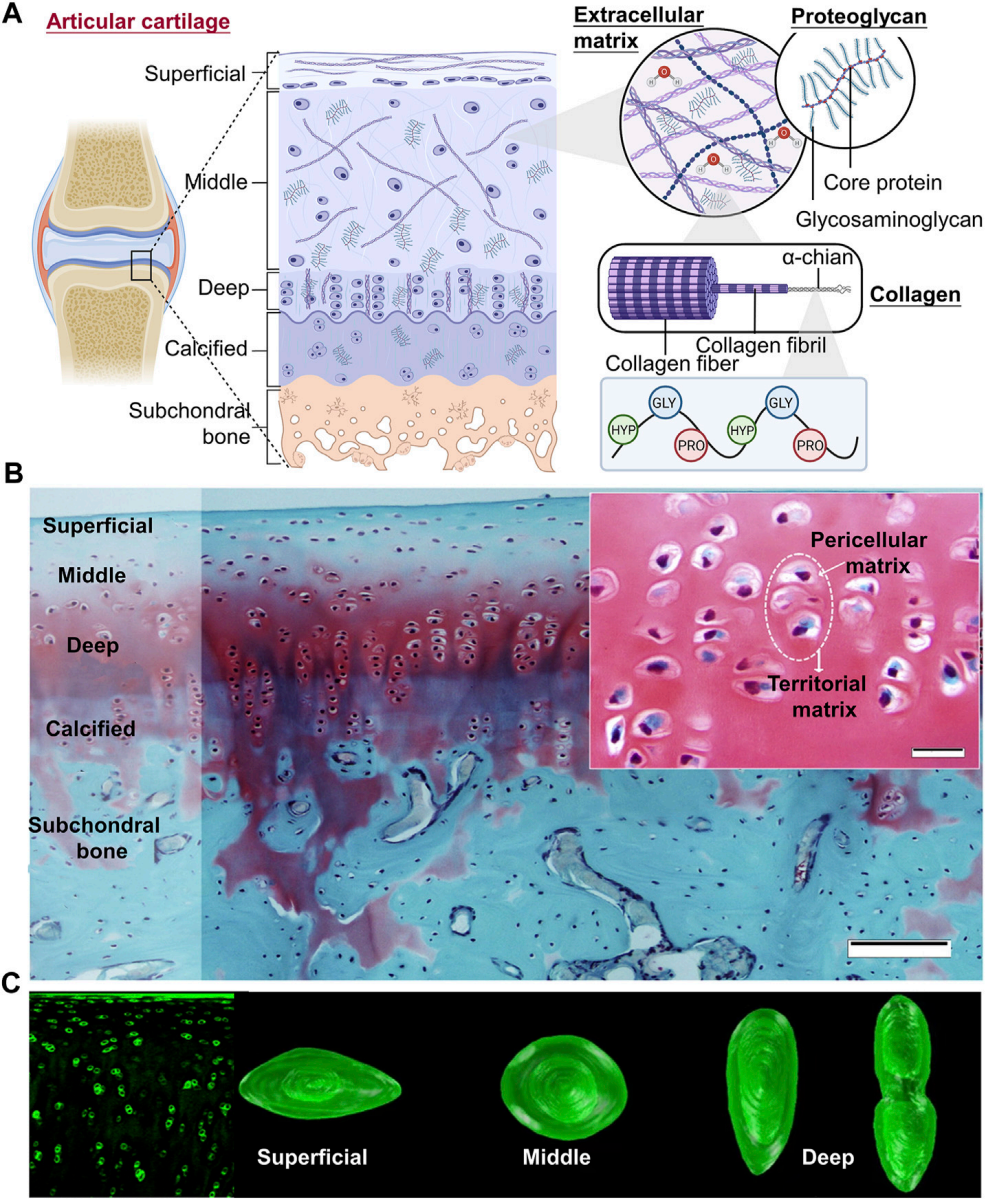
Three overlapping zones of articular cartilage **(A)** Three overlapping zones from the surface to the subchondral bone can be found in adult AC: the superficial zone (SZ), the intermediate zone (IZ), and the deep zone. Within these zones, the morphology of chondrocytes, as well as the content and architecture of ECM, reflect the forces exposed during movement (Figure created using BioRender.com). **(B)** Knee joint cartilage structure in rabbits. Articular cartilage is organized into zones in this representative Safranin O/Fast Green-stained tissue segment from the rabbit knee joint. Scale bar: 100 µm. The cartilage ECM regions are highlighted in the little image. Scale bar: 20 µm (Armiento et al., 2018). **(C)** (Left) Blocks of cartilage tissue immunolabeled for type-VI collagen are shown in fluorescence confocal pictures. (Right) In each zone, chondrons showed noticeable variations in height, shape, and volume (Choi et al., 2007).

The cartilage in the joints is incredibly sensitive to mechanical strain. The mechanical environment primarily determines the structure of cartilage, which may play a role in cartilage mechanoadaptation (Vincent and Wann, 2019). Three overlapping zones that move from the surface to the subchondral bone make up adult AC: the superficial zone (SZ), the intermediate zone (IZ), and the deep zone (Figure 2) (Armiento et al., 2018; Hodgkinson et al., 2022). Within these zones, the morphology of chondrocytes, as well as the content and architecture of ECM, reflect the forces experienced during movement. In the superficial zone, chondrocytes are immature, have an oblate form, and are scattered singly on the cartilage surface. To distribute shear pressures during articulation, type II collagen fibers are oriented transversely. In the intermediate zones, chondrocytes show a somewhat rounded shape, hypertrophy, and cluster together. Type II collagen fibers are distributed randomly to withstand the pressure coming from various directions since this zone is subject to compressive and shear stresses. In contrast, the pericellular matrix consisting of collagen VI, which surrounds the chondrocytes in the deep zone, is collectively referred to as a chondron. To resist compressive pressures, collagen II fibers are thick and parallel to the joint surface (Figure 2) (Yu et al., 2023), and high proteoglycan concentrations encourage water retention (Wang Z. et al., 2022; Hodgkinson et al., 2022). Col II, PRG4, and FGF content decrease as cartilage sites become deeper; the opposite is true for Col X and glycosaminoglycan (GAG) content (Wang Z. et al., 2022). Structural heterogeneity is more conducive to chondrogenesis. The calcified cartilage zone (CCZ), the transition area between cartilage and subchondral bone, also plays a major role in the repair process after osteochondral defects. At 24 weeks after surgery, the cartilage layer of the CCZ group was primarily repaired by hyaline cartilage, in contrast to the defects in the blank control and non-CCZ groups, which were filled with fibrous tissue. The experiment explored the feasibility of using trilayer scaffold containing natural CCZ as an intervention in osteochondral tissue engineering (Huang Y. et al., 2021). A study determined that spatial distributions of heterogeneous mechanical stimuli might affect the cell behavior. Cell viability is low close to the porous compression-platen interface, although it rises with depth, according to cross-sectional investigations (Kisiday et al., 2009).

## 3 Classification and the influence of mechanical stimuli

The expression of MSC chondrogenic genes and the formation of cartilage are both influenced by mechanical stimulation. The maintenance of the homeostasis of articular cartilage is aided by the chondrocyte’s responses to suitable mechanical stimulation (Le et al., 2020). However, inappropriate mechanical stimulation causes cell death (Healy et al., 2008). Mechanical stimuli are mainly categorized into four groups: mechanical compression, fluid shear stress, hydrostatic pressure, and osmotic pressure; these are applied singularly or in combination with others.

### 3.1 Mechanical compression

For cartilage remodeling, mechanical compression has both advantages and disadvantages. Similar metabolic response to compression can be seen in animal research and *in vitro*. While dynamic compression can considerably increase matrix production, static compression significantly reduces the synthesis of collagen and PGs (Alizadeh Sardroud et al., 2021).

Temporal cues controlled by the cell-autonomous circadian clock are one of the many external elements that influence chondrogenesis. A recent study, for the first time, demonstrated that optimal mechanical compression directly enhances cartilage matrix production by entraining the molecular clockwork in chondroprogenitor cells. The chondrogenic markers SOX9 and ACAN, in addition to the several core clock genes and proteins, also exhibited a consistent sinusoidal rhythmic expression pattern. The results showed that the synchronized, rhythmic expression of the chondrogenic transcription factors and main circadian clock genes, at least partially facilitated increased chondrogenesis brought on by mechanical compression (Vágó et al., 2022). This experiment combines mechanical factors with biological cycle factors and proves their correlation.

**FIGURE 3:**
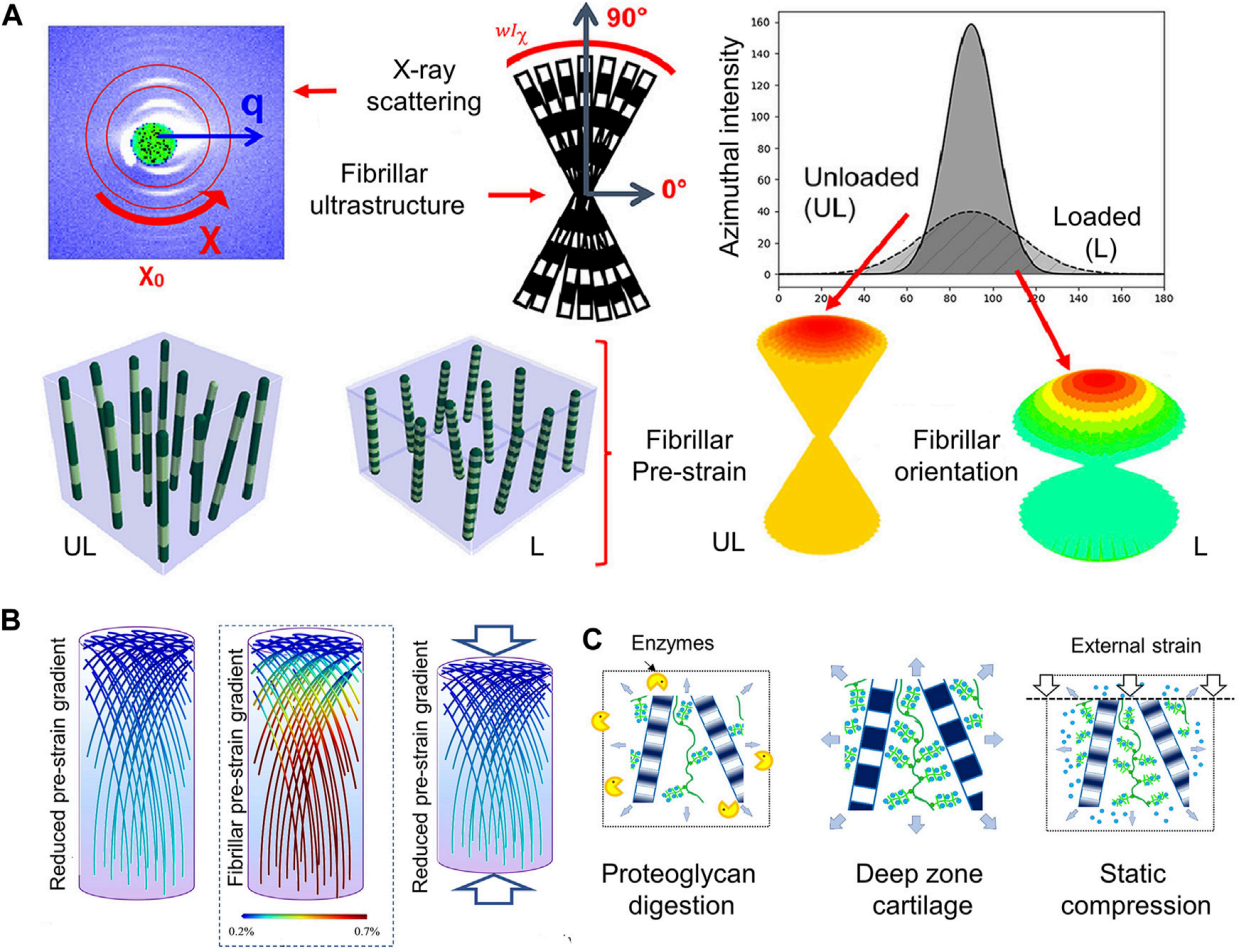
The structure of collagen fibrils reflecting the mechanical microenvironment **(A)** Utilizing X-ray scattering and fibrillar ultrastructure explores structural response of the Type II collagen fibrils in cartilage to cyclic loading. Axially symmetric broadening of the fibril orientation distribution and reduction of fibril pre-strain (at a fixed orientation) are shown. The fibril angle with respect to the sample (and loading) axis is shown using color-coding: Green fibrils are off-axis in relation to the joint surface, and red fibrils are close to vertically oriented fibrils at 90° to the joint surface (Inamdar et al., 2021). **(B)** (Centre): Based on scanning SAXS measurements, it has been determined that the articular cartilage exhibits a new gradient of rising fibrillar pre-strain from the surface/transitional (upper) zone to the deep (lower) zone. Pre-strain levels are indicated via a color scale on the fibrils, with blue denoting low pre-strain and red denoting high pre-strain (color online). In a way similar to (Left): treatment with chondroitinase sulphate to remove some of the PG phase, compression (Right) lowers the fibrillar pre-strain gradient (color gradient lowers) (Inamdar et al., 2019). **(C)** Ultrastructural mechanism enabling these fibrillar gradients: enzymatic (left) and load-induced (right) changes to the original fibrillar nanostructure (center) result in loss of water molecules originally bound to PGs, thus reducing the internal pre-stress exerted on the fibrillar network by the PG phase (Inamdar et al., 2019).

It is thought that articular cartilage’s collagen fibrillar architecture undergoes biomechanical modifications that are essential for facilitating appropriate joint function. The mechanisms behind cartilage’s capacity to endure prolonged repetitive compression as well as the structural response of Type II collagen fibrils to cyclic stress *in situ* have been highlighted. The fibrillar response including inter-fibrillar variability orientation, and fibrillar strain were measured by synchrotron small-angle X-ray scattering (SAXS), and 3D reconstruction techniques. The study showed that the fibrils reversibly alter the width of the fibrillar orientation distribution and reduce fibrillar pre-strain under repeated cyclic loading (Figure 3A) (Inamdar et al., 2021).

The functionality of biological tissues and the prevention of harmful interfacial stress concentrations depend on the biomechanical gradients, which means a depth-dependent variation in some mechanical parameters. Periodic fibrillar banding (D-period), a sensitive indicator of zonal structure to the mechanical environment at the nanoscale, is one of the main parameters. This experiment also explored the relationship between proteoglycans and the microstructure of collagen. The removal of extrafibrillar proteoglycans causes fibrillar alterations that are similar to static compression (Figures 3B,C) **(** Inamdar et al., 2019).

However, the response to dynamic compression relies on the frequency, amplitude (Xie et al., 2006; Uzieliene et al., 2023), zone (Jeon et al., 2012), time-point (Ge et al., 2021; McDermott et al., 2021), and occurs in combination with biochemical cues (Sani et al., 2022; Li et al., 2023).

Xie et al. (2006) showed that when chondrocytes were seeded in the biodegradable elastomeric scaffold poly (L-lactide-co-caprolactone) (PLCL) at 10% compressive strain and 0.1 Hz, the level of type II collagen mRNA expression was elevated (Xie et al., 2006). The molecular mechanism underlying this process was also investigated. The area between −509 and −109 base pairs, where the transcription factor Sp1 is found, is where the short promoter responds to continuous dynamic compression most actively, according to a mutant deletion investigation. Additionally, it was demonstrated that mechanical compression activates transcription, possibly via the Sp1 binding sites in the proximal area of the *COL2A1* gene promoter, to raise the level of type II mRNA expression (Xie et al., 2006). In contrast, a different study demonstrated that compressed OA cartilage under strong mechanical pressure had lower overall levels of glycosaminoglycans and proteoglycans (Uzieliene et al., 2023).

Patients with osteoarthritis (OA) who have zonal chondrocytes can benefit from tailored compressive stimulation since different zones of articular cartilage react differently to compressive loading: Only superficial constructs displayed greater PRG4 staining, retained more GAG (P 0.01), and developed higher compressive moduli than unloaded controls (Jeon et al., 2012).

A 2021 study explored how mechanical dynamic compression affected the chondrogenic development of mesenchymal stem cells (SMSCs) generated from human synovium. The scientists discovered that, in comparison to the unloaded control, dynamic compression that was started at an early time point decreased the expression of markers unique to chondrocytes and hypertrophy. In contrast, dynamic compression applied at a later time point improved the cartilage matrix’s levels of gene expression while suppressing SMSCs’ hypertrophic growth when compared to unloaded controls (Ge et al., 2021). The results imply that dynamic mechanical compression loading at optimal time-point is essential for maintaining the cartilage phenotype and for promoting SMSCs’ chondrogenic development. A different study, investigated the effect of the chondrogenic priming duration, showing that dynamic compression for certain duration enhanced the engineered tissue’s equilibrium and dynamic modulus. Dynamic compression improved the expression of *COL2A1* and *ACAN* mRNA at the conclusion of the loading phase for priming durations of 2 weeks or longer. According to the authors, loads start at priming durations of 4 weeks or fewer suppressed transient osteogenic signaling (*RUNX2, OPN*), as well as the expression of *CYR61*, a gene that is a target of the YAP/TAZ-TEAD pathway (McDermott et al., 2021).

According to one study, mechanical stimulation showed no negative effects on the viability and growth of cells. It decreased the expression of matrix metalloproteinase-3 (MMP-3) and increased the expression of chondrogenic markers such as aggrecan, proteoglycan-4, and collagen type II. Despite showing modest increase in neocartilage development, IGF-1 was not as effective as mechanical stimulation (Sani et al., 2022).

Different biological factors may have opposite effects on endochondral ossification and cartilage regeneration. For the dual regulation of endochondral ossification, a coordinated dynamic mechanical stimulation is paired with various biochemical cues, such as parathyroid hormone and hydroxyapatite in the outer and inner region, respectively. In particular, dynamic mechanical stimuli combined with parathyroid hormone in the outer region prevent endochondral ossification and lead to cartilage regeneration, while dynamic mechanical stimulus combined with hydroxyapatite in the inner region encourages endochondral ossification and produces effective subchondral bone regeneration (Li et al., 2023).

### 3.2 Fluid shear stress

Shear stress is a contentious technique since it can sometimes cause cell death (Healy et al., 2008), but other times it can encourage tissue regeneration (Gemmiti and Guldberg, 2006; Valonen et al., 2010; Gonçalves et al., 2011).

When tissue is compressed, about 70% of the water is released, which could cause fluid shear stress at or near the cellular membrane. When the tissue is unloaded, osmotically, the water is sucked back.

Cartilage is a highly aquiferous connective tissue. When the tissue is compressed, about 70% of the water is released, which could cause fluid shear stress at or near the cellular membrane. When the tissue is unloaded, osmotically, the water is sucked back (Sharifi and Gharravi, 2019). Therefore, when water is relocated during mechanical compression, the chondrocytes may undergo fluid shear stress.

Static cultures are not widely used because shear stress has been associated with the activation of pro-inflammatory and pro-apoptotic proteins in chondrocytes. Shear stress has been associated to matrix degradation in cartilage tissue. When chondrocytes experience shear stress from mechanical loading, an important proinflammatory enzyme called cyclooxygenase-2 (COX-2) is produced (Healy et al., 2008). However, certain shear stress magnitudes have been found to be advantageous (Gemmiti and Guldberg, 2006; Valonen et al., 2010; Gonçalves et al., 2011). In comparison to static culture, dynamic fluid flow culture has a number of benefits, including improved mass transport and a more regulated biochemical environment.

To establish the ideal shear stress levels for the development of new tissues, an adjustable device was created. The mechanical characteristics of neocartilage *in vitro* were subsequently improved up to 3.6-fold as a result of the discovery of a favorable window of fluid-induced shear (FIS) stress. It was discovered that stress of 0.05–0.21 Pa considerably improved build properties. A mechanistic investigation was conducted to better understand the positive effects of FIS stress, and the results showed that the primary cilia of chondrocytes are mechanically gated complexes that are triggered by FIS stress (Salinas et al., 2020). The advantages of FIS stress are not restricted to the neocartilage’s surface. Top to bottom cross-sections were stained histologically. The findings demonstrated that the fiber density seen on the neocartilage constructs’ surfaces was constant inside the construct (Salinas et al., 2020).

Abnormal mechanical stimuli are related to the pathophysiology of articular joints and aberrant fluid shear stress (FSS) greatly reduces chondrocyte survival and causes widespread disruption of cell shape. High quantities of inflammatory mediators are produced when FSS is abnormal, which causes cartilage to degeneration and deteriorate (Jin et al., 2021). Gene expression profiles related to osteoarthritis are recapitulated by prolonged application of high FSS to chondrocytes. The information points to a possible connection between the pathophysiology and development of OA and the exposure of chondrocytes and cartilage to aberrant mechanical loading (Zhu et al., 2010). Another study measured the FSS in the interstices surrounding the chondrocytes in growth plate cartilage, demonstrating that the FSS, which is caused by fluid flow over the cell surface, may have the ability to stimulate chondrocytes in the reserve zone close to the subchondral bone plate interface (Kazemi and Williams, 2021).

**FIGURE 4:**
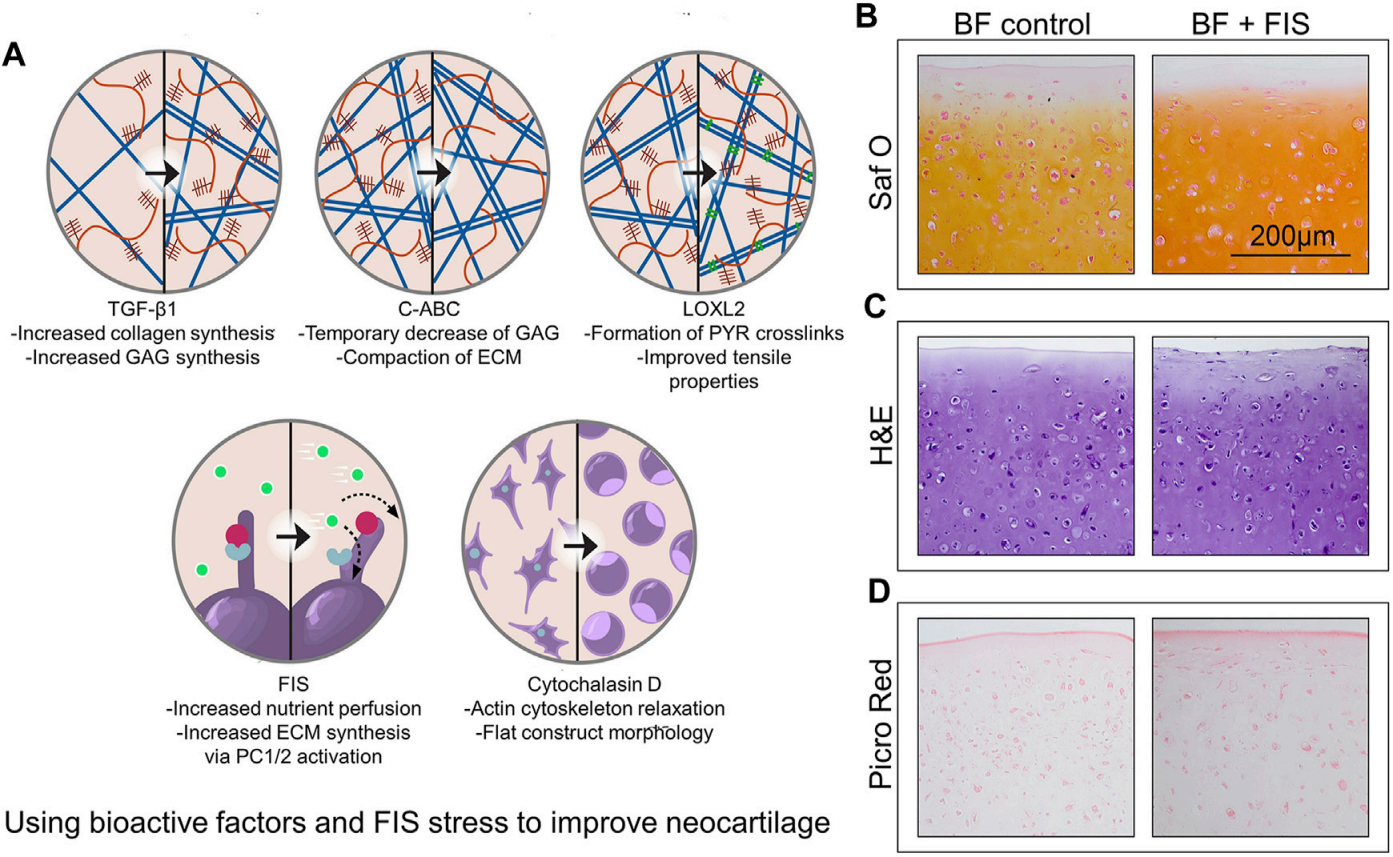
Using bioactive factors and FIS stress to improve neocartilage **(A)** Exogenous TGF-1 and FIS stress may enhance ECM content in a comparable way. Further research and combination with other mechanical stimulation regimens are encouraged in the hopes of further improving the functional properties of neocartilage by studies about additional bioactive factors, such as transforming growth factor beta 1 (TGF-β1), chondroitinase ABC (C-ABC), lysyl oxidase-like 2 (LOXL2), polycystin 1/2(PC1/2), and cytochalasin D (Salinas et al., 2022). **(B–D)** The intensity of glycosaminoglycan staining is increased when bioactive substances and FIS stress are combined. The examples show representative images of glycosaminoglycan content **(B)**, general cellular and tissue morphology **(C)**, and general collagen content **(D)**. Bioactive factors (BF), fluid-induced shear (FIS), hematoxylin and eosin (H, E), Picrosirius Red (Picro Red), and safranin O(Saf O) are among the abbreviations used (Zhang J. et al., 2021).

It has been demonstrated that the primary cilia, which can react to the FIS stress (Salinas et al., 2020), regulate TGF-signaling (Moore and Jacobs, 2018). One work showed that the species origin of chondrocytes and the addition of bioactive factors all affect the aggregate modulus of neocartilage stimulated by FIS stress. The research demonstrated the effect of FIS stress stimulation across sources of bovine and minipig cells. In addition, when FIS stress was added to bioactive factors, advancement in mechanical and biochemical properties was evident, with increased shear modulus by 115% compared to bioactive factor-only controls (Figure 4A) (Salinas et al., 2022). Histologically, samples treated with bioactive substances and FIS stress showed stronger Saf O staining, which is a sign that the neocartilage structures contain more glycosaminoglycans (Figure 4B), and similar staining intensities using H&E (Figure 4C). The spatial arrangement of collagens within the matrix is visible by Picro-Sirius red staining. FIS stress elevated peripheral staining, indicating higher collagen deposition on the outside margins of the neocartilage constructs, despite the groups appearing to have equal overall intensities (Figure 4D). Cells are typically not exposed to fluid mechanical signals after bioprinting, such as FSS, which are essential for tissue formation and function in both health and illness. The bioreactor acts as a simulator to help with the *in vitro* maturation of 3D cell-laden scaffolds for the creation of synthetic human tissues (Zhang J. et al., 2021).

Increased nutritional perfusion and waste transfer may be the cause of FIS stress-induced benefits, but they may also be the result of intricate cellular signaling processes and mechanotransduction-driven matrix remodeling (Salinas et al., 2020). The hydrodynamic environment of the new bioreactor, which is subject to FIS stresses and improved mass movement, may be a successful functional tissue-engineering approach for enhancing matrix composition and mechanical characteristics *in vitro*.

### 3.3 Hydrostatic pressure

The choice of HP experimental variables can have a big impact on how manufactured cartilage develops. Based on the following variables: static or dynamic, pressure magnitude, and experiment time, the different effects of various HP regimes on proteoglycan synthesis were examined. Analysis showed that the use of the static HP, a magnitude within the mid-high physiological range of cartilage (5–10 MPa), and a research duration of 2 weeks or longer most likely resulted in a strong anabolic response (Hodder et al., 2020). However, excessive HP is a major factor in the development of articular cartilage disease, such as OA. After HP loading, high levels of Gremlin-1, a mechanical loading-inducible factor in chondrocytes, are found in the middle and deep layers of cartilage tissue (Chang et al., 2019).

Although there is not a single best practice that can be applied to all culture systems, hydrostatic pressure (HP) is arguably one of the most important mechanical stimuli for cartilage. Mechanobiology, a vast field of study, is where the effects of HP on cartilage development reside. Interstitial fluid is pressured during cartilage loading, and the surrounding matrix prevents pressure loss by slowing the fluid flow rate from pressurized areas. HP is the term for this type of fluid pressurization, which results in homogenous stress all around the cell without cellular deformation (Pattappa et al., 2019). HP has been extensively used as an agent for promoting the differentiation of MSCs (Wagner et al., 2008), cartilage formation (Chen et al., 2017), and integration between the host cartilage milieu and the regenerated cartilage (Cheng et al., 2019) in tissue engineering.

With the hydrostatic pressure loading regime, chondrogenic genes such as *ACAN, COLIIA1*, and *Sox9* exhibit a substantial increase in mRNA expression. An earlier work showed that HP promoted MSC differentiation when multipotent differentiation factors were present *in vitro*. This finding raises the possibility that the HP regime may be crucial for cartilage growth and regeneration *in vivo* (Wagner et al., 2008). A cartilage regeneration model-based 3D tissue culture *in vitro* was used in one investigation. It was discovered that HP from the newly created bioreactor effectively increased the development of 3D cartilage by enhancing its mechanical strength, thickness, and uniformity (Chen et al., 2017). The study therefore provides an essential approach for improving cartilage regeneration *in vitro*.

In rabbit temporomandibular joints, it was discovered that BMSCs sheet fragments and platelet-rich fibrin granules transplanted into feasible HP-pretreated constructs improved the integration between the regenerated cartilage and host cartilage milieu, and achieved boundary-less repair between the residual host cartilage and the neocartilage (Cheng et al., 2019).

Using 3D bioprinting, it is possible to create constructions that mimic the mechanical characteristics of the natural articular cartilage by adjusting the HP in cartilage tissue. According to Finite Element (FE) modeling, the reinforcement of interpenetrating polymer network (IPN) hydrogels with particular polycaprolactone networks reduced radial expansion and increased the HP produced within the IPN when compressive loading was applied (Schipani et al., 2020).

**FIGURE 5:**
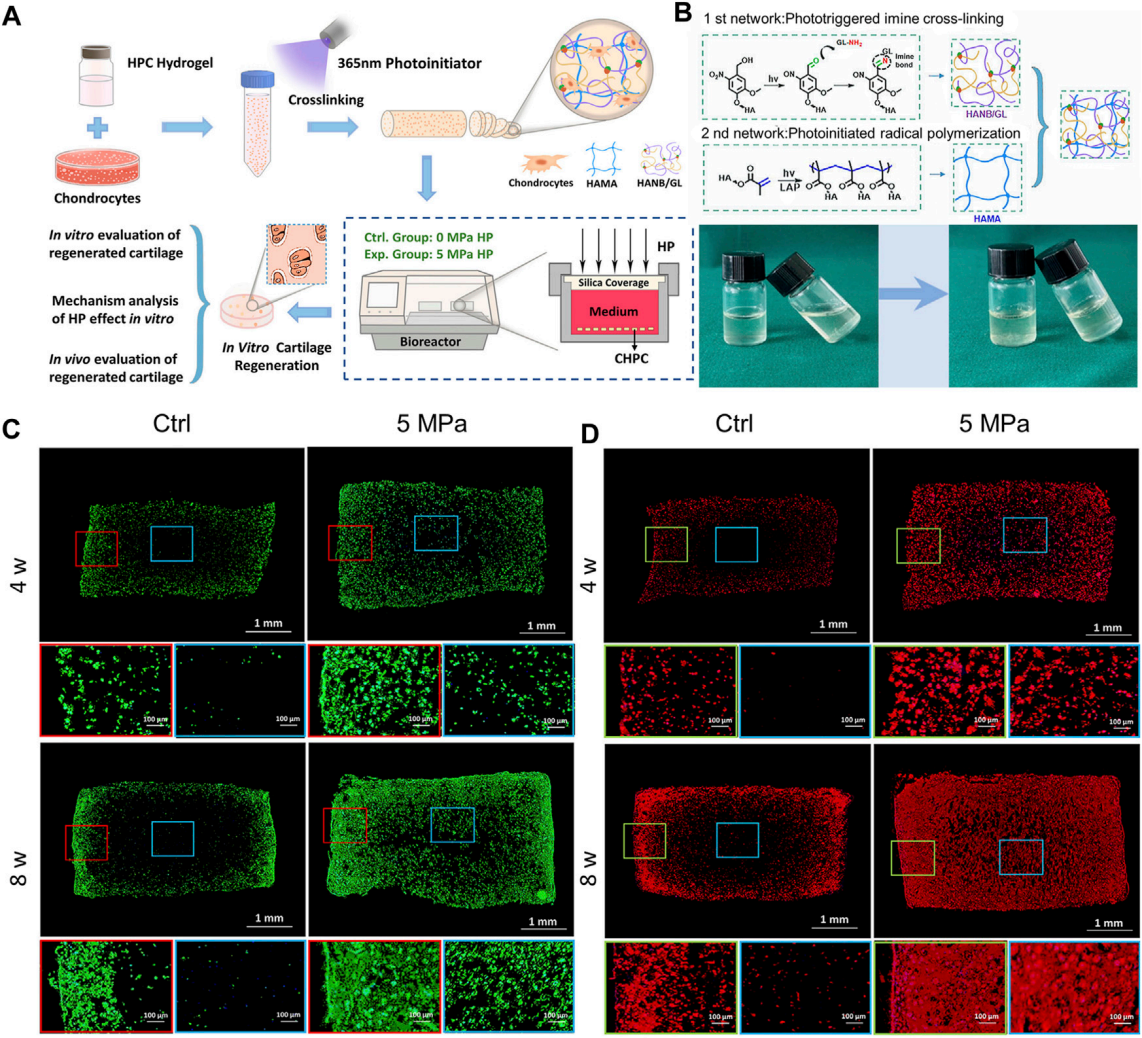
A hybrid photocrosslinkable hydrogel-based hydrostatic pressure (HP) bioreactor that controls cartilage regeneration **(A)** Diagrammatic representation of chondrocyte laden HPC hydrogels (CHPC)-based *in vitro* cartilage regeneration in an HP bioreactor. **(B)** (Top): Diagram of hybrid photocrosslinkable (HPC) hydrogel building process and double-network construction. (Bottom): Pre- and post-cross-linking images of the HPC hydrogels using light irradiation technique. **(C, D)** Phalloidine and Collagen II of CHPC produced *in vitro* were stained using immunofluorescence. Blue frames represent the inner regions, while red and green frames represent the periphery (Zhao et al., 2022).

No effective reports of *in vitro* cartilage regeneration based on photocrosslinkable hydrogels have been made due to nutrient absorption barriers brought on by dense networks and static culture conditions. To address this issue, a hybrid photocrosslinkable hydrogel was controlled using HP given by the bioreactor (Zhao et al., 2022). According to a 2022 study, HP completely counteracted the negative effects of hybrid photocrosslinkable (HPC) hydrogels at 3% weight percentage; significantly increased cell viability, proliferation, and ECM deposition by improving nutrient transportation and up-regulating the expression of genes specific to cartilage; and successfully regenerated homogeneous cartilage with a thickness of over 3 mm. While few living chondrocytes were seen in the center of the control group, chondrocyte-laden HPC hydrogels (CHPC) in the HP group had many live cells throughout, according to phalloidine fluorescence labeling. The distribution of type II collagen in the HP group was clearly denser at both 4 and 8 weeks than in the control group, whose center areas were nearly empty (Figure 5) (Zhao et al., 2022).

### 3.4 Osmotic pressure

Osmotic pressure sometimes causes chondrocytes to burst (Hara et al., 2018) and, also be used to enhance the mechanical properties of cartilage tissue engineering grafts (Schuiringa et al., 2023).

Osmotic pressure was cited in another study as an external component that could cause chondrocytes burst. It has been demonstrated that osmotic pressure can influence chondrocyte morphology. Mechanical stress and hypotonic solutions both greatly lead to chondrocyte burst. Hara et al. additionally proposed that chondrocyte burst can be connected to the creation of space for mineral expansion (Hara et al., 2018).

Osteoarthritis results from cartilage abnormalities. Due to their capacity to imitate the natural ECM, hydrogels offer a viable regenerative approach for addressing such abnormalities. Hydrogels that are frequently utilized for tissue regeneration, however, are too supple to withstand load-bearing in the joint. To address this problem, researchers have created an implant in which the osmotic pressure created by a charged hydrogel’s swelling potential, which is constrained from swelling by a textile spacer fabric, serves as the mechanical load-bearing function (Schuiringa et al., 2023).

Joint-loading and weight-bearing strains compress articular cartilage, which is then followed by a tissue bulge during off-loading. Osmotic pressure is affected by changes in water content, which are brought on by this loading and unloading. Water is expelled from the tissue during compressive loading, which raises the concentration of local proteoglycan and puts chondrocytes under hyperosmotic stress. Surface, middle, and deep zones make up the longitudinal depth of articular cartilage. Each zone has a varied amount of osmotic pressure since each one is made up of distinct extremely negative ions. Previous researches have demonstrated the sensitivity of chondrocytes to both hyper- and hypo-osmotic alteration, which affects cell shape (Erickson et al., 2003; Turunen et al., 2012; Wang et al., 2015), metabolism (Hopewell and Urban, 2003; Tew et al., 2009) and biomechanics (Erickson et al., 2003; Wang et al., 2015). The rate of the osmotic challenge determines how the chondrocytes react to osmotic pressure (Wang et al., 2015), the integrity of the cartilage (Turunen et al., 2012) and the phenotype of the chondrocytes (Tew et al., 2009). Osmolarity also determines *in vitro* chondrogenic differentiation (Liang et al., 2012; Caron et al., 2013).

Spheroidal cartilage organoids produced by particular chondrocytes from the longitudinal depth zone were cultured at various osmotic pressures. Takada et al. discovered that all zone-derived chondrocytes significantly enhanced the transient expression of ACAN and collagen type-II (Takada and Mizuno, 2018).

In another investigation, the morphology and biomechanics of chondrocytes were evaluated in response to abrupt and progressive hypo-osmotic pressure (Wang et al., 2015). The 66% of chondrocytes showed an increase in diameter followed by a regulatory volume decline (RVD) in response to abrupt hypo-osmotic stress, while 25% showed no RVD. On the other hand, cells that had gradually experienced hypo-osmotic stress showed decreased cell enlargement without a subsequent RVD. For cells exposed to abrupt hypo-osmotic stress, the equilibrium modulus increased. The gradual hypoosmotic challenge, however, had no effect on the mechanical characteristics of the chondrocytes (Wang et al., 2015). It is demonstrated that the rate of the hypo-osmotic challenge has a significant impact on the morphology and biomechanics of chondrocytes.

**FIGURE 6:**
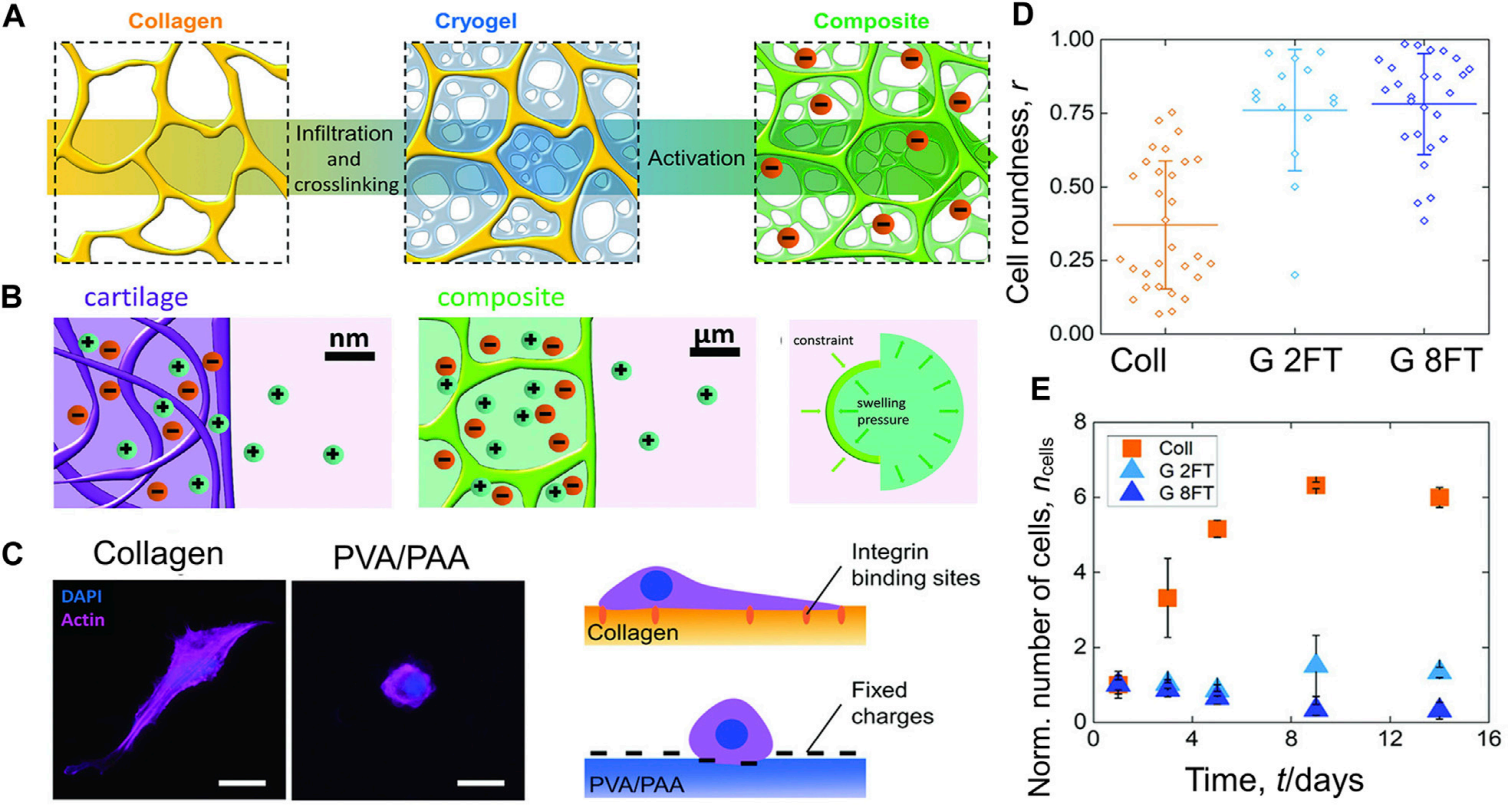
The composites’ mechanical behavior is influenced by electrostatic and osmotic forces **(A)** A freeze-dried collagen scaffold is infiltrated with a PVA and PAA solution and then cross-linked by freezing and thawing. The created composite is then activated at a high pH. **(B)** (Left and middle): The composite materials replicate the ionic environment seen in genuine articular cartilage. Due to the existence of negative fixed charges, the concentration of positive counter-ions is higher than in the surrounding solution. (Right): Ionic imbalance induces osmotic swelling pressure. **(C)** (Left): Fluorescence microscopy was used to examine the interactions between materials and cells, which were planted on top of in 2D components. (Right): Cell-materials interaction and consequent morphology are shown schematically. **(D)** The ratio of the cell’s roundness ranges from 0 (linear shape) to 1 (circular shape). **(E)** Over the course of 14 days of culture, the number of cells on the 2D components has stabilized relative to day 1 and error bars show the standard deviation (Offeddu et al., 2018).

Composite materials based on a polyelectrolyte hydrogel embedded in a collagen scaffold mimic the specific molecular interactions of cartilage (Figure 6A). These composites have a mechanical structure similar to articular cartilage that is influenced by osmotic and electrostatic function. Positive counterions between the material and the ambient solution are uneven as a result of the charged matrix’s restriction. The resulting imbalance creates an osmotic swelling pressure (Figure 6B). Collagen or poly (vinyl alcohol)-poly (acrylic acid) hydrogels were used to create 2D films that were cross-linked in the same way and to the same degree as the 3D scaffolds in order, to better view and understand the interaction between cells and the components of the composites. After two and eight freeze-thaw cycles, the materials for the composites (C) and cryogel (G) components, respectively, were evaluated (Figures 6C–E) (Offeddu et al., 2018). The biomimetic materials described here represent a completely new technique of composites that are osmotically stiffened.

## 4 Mechanisms involved in the transduction of macroscopic mechanical forces into intracellular events

Articular cartilage is one of the most important weight-bearing parts of the human body. Therefore, the chondrogenic differentiation of stem cells is influenced by many intracellular and extracellular mechanical signals. Stem cells and chondrocytes can sense and respond to various mechanical signals through a series of mechanisms. Mechanoreceptors, which are the first responders to mechanical forces, include primary cilia, integrins, ion channels, etc. These mechanoreceptors convert macroscopic mechanical signals into specific chemical signals, which are transmitted through a variety of complex downstream pathways to affect the expression of related transcription factors, and ultimately produce biological effects to affect cell behavior.

### 4.1 Mechanoreceptors

Chondrocytes react to mechanical stimuli “directly” via PCM deformation, cell-ECM adhesions (primary cilia, integrin), and cell sensory structures (ion channels), or “indirectly” as a result of the release of sequestered growth factors and their interactions with cell receptors.

#### 4.1.1 Primary cilia

The axon section and the basal body make up primary cilia, which are organelles that project from the cell surface into the ECM. The basal body of the primary cilia anchors to the inner cell membrane, while the axon section of the cilia protrudes from the membrane’s surface. The basal body, a modified version of the centriole, is the source of primary cilia, which are microtubule-based organelles (Tao et al., 2020). The primary cilium’s roles in the articular cartilage are as an antenna to sense the biomechanical environment, control the secretion of ECM components, and store cellular positioning information. Numerous ion channels and signaling receptors are found in primary cilia (McGlashan et al., 2006; Lee et al., 2015).

Using immunofluorescence and confocal imaging, the ECM receptors on chondrocyte plasma membranes and the main cilia have been examined. On the plasma membrane, every receptor that was tested showed a punctate distribution. On the primary cilia, integrins α2, α3, β1, and NG2 were also visible (McGlashan et al., 2006). This study is the first to show that integrins and NG2 are expressed on primary cilia in chondrocyte. Using a FRET-based biosensor fused to ARL13B, Lee et al. report the first observations of Ca^2+^ signaling within primary cilia in osteocyte. They demonstrated that fluid shear stress causes Ca^2+^ increases in primary cilia of osteocytes, which are dependent on both intracellular Ca^2+^ release and external Ca^2+^ entry (Lee et al., 2015).

**FIGURE 7:**
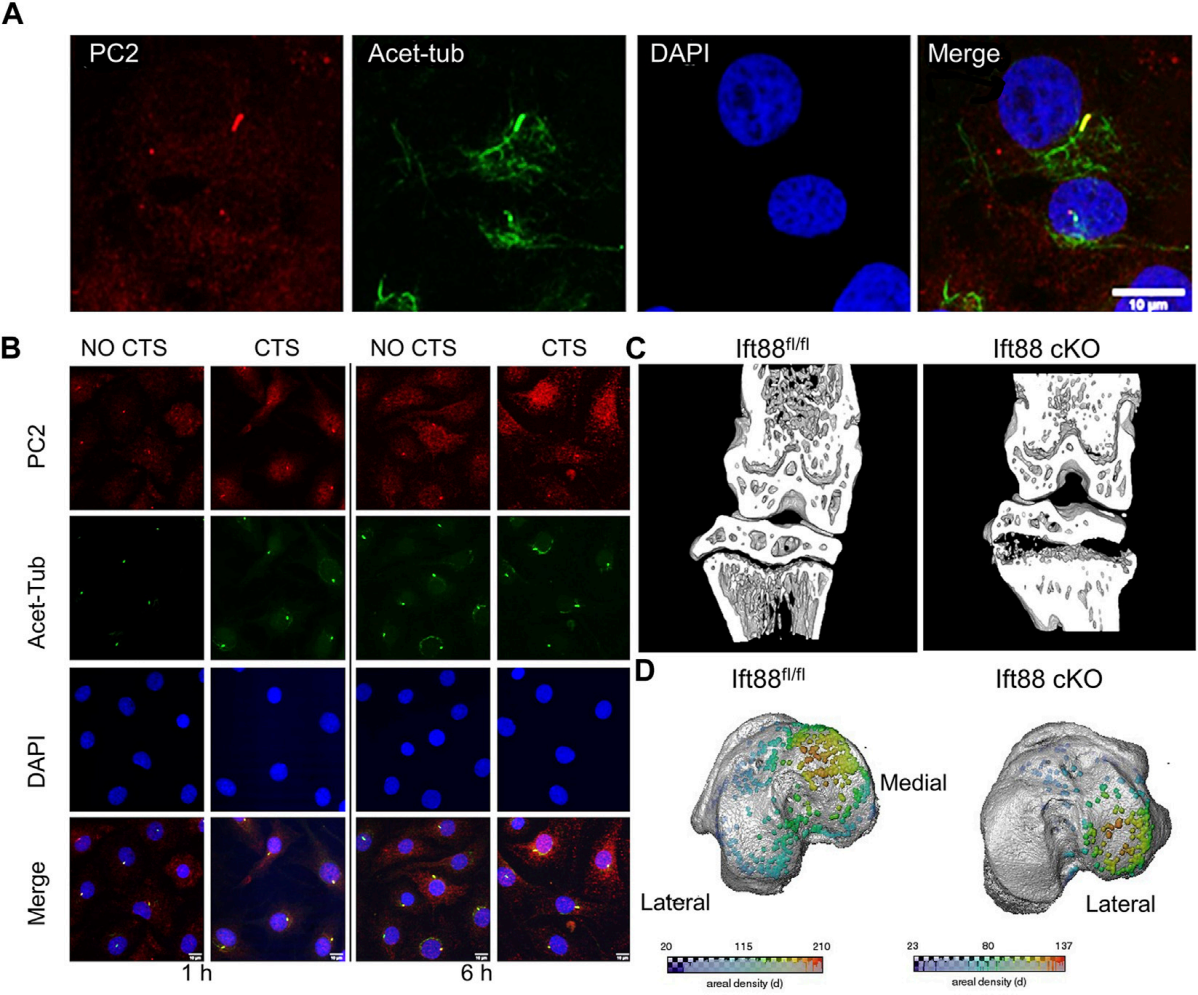
Different expression of primary cilia mediated by mechanical stimulation **(A)** Primary cilia were immunolabeled for acetylated tubulin (acet-tub, green), bovine chondrocytes were labeled for polycystin-2 (PC2, red), and primary DAPI was used to counterstain the nuclei. Scale bar: 10 µm (Thompson et al., 2021). **(B)** To explore the effect of cyclic tensile strain (CTS), chondrocytes were stained with DAPI (blue), primary cilia were stained with acetylated -tubulin (green), and polycystin-2 (PC2) was used to mark chondrocytes. Scale bar:10 µm (Thompson et al., 2021). **(C)** Micro-CT partial 3D construction of 10 weeks mice to explore the effect of the deletion of intraflagellar transport protein 88. The effects of IFT88 deletion are only felt in the growth plate’s periphery, just below the knee’s load-bearing articular surfaces (Coveney et al., 2022a). **(D)** Growth plate bridges across the knee’s tibial articular surfaces are mapped using three dimensions representation. The density of the bridges is shown by the color scale (Coveney et al., 2022a).

As one of the transient receptor potential polycystic of ion channels, polycystin-2 (PC2) and polycystin-1 (PC1) have been linked to cilia-mediated mechanotransduction in epithelial cells. Uniaxial cyclic tensile strain (CTS) was used to mechanically stimulate isolated chondrocytes in order to study the impact on PC2 ciliary location and matrix gene expression. The cilium was discovered to have a higher level of PC2 localisation, which co-localized with the ciliary marker acetylated -tubulin (Figure 7A). Response to mechanical stimulation results in an increase in PC2 ciliary localisation (Figure 7B) (Thompson et al., 2021). Furthermore, mechanical stimulation from the ECM can change the degree of deflection (Jensen et al., 2004), length (Fu et al., 2019; Zhao et al., 2020), and orientation (Farnum and Wilsman, 2011) of primary cilia, indicating that primary cilia can sense mechanical signals in bone formation and growth.

The ciliary axoneme was visible interdigitating between collagen fibers and condensed proteoglycans using tomography and TEM. The primary cilium is bent as a result of mechanical stimuli conveyed through matrix macromolecules, suggesting that it may function as a mechanosensor for skeletal patterning and growth (Jensen et al., 2004). Primary cilia in chondrocytes are sensitive to mechanical forces, and when subjected to cyclic tensile strain or hyperosmotic stresses, their length decreases dramatically. The principal cilial length in healthy cartilage is 1.5 mm in the deep layer and 1.1 mm in the superficial layer (Zhao et al., 2020). Treatment with IL-1β at 1 ng/mL produced a statistically significant increase (of 14%) in cilia length. However, this effect was completely inhibited under mechanical loading (Fu et al., 2019). Additionally, there were differences between load-bearing and non-load-bearing zone in the direction of the chondrocyte primary cilia. The axoneme extends from the cellular surface towards the subchondral bone in load-bearing areas of the superficial zone. This uniformity disappears in areas not supporting loads (Farnum and Wilsman, 2011).

During skeletal development, primary cilia proteins, which are involved in the transduction of biological and physiochemical signals, regulate the maturation of cartilage. Researchers tested the effects of the ciliary protein intraflagellar transport protein 88 (IFT88) on postnatal cartilage in mice with the Ift88 gene conditionally knocked out (Ift88-KO). The findings show that IFT88 acts as a chondroprotector in articular cartilage by preventing cartilage from calcifying by maintaining a Hh signaling threshold under physiological loading (Coveney et al., 2022b).

Coveney et al. used a cartilage-specific, inducible Cre (AggrecanCreERT2 Ift88fl/fl) to conditionally target the ciliary gene intraflagellar transport protein 88 (Ift88fl/fl) in the juvenile and adolescent skeleton, in order to investigate the role of primary cilia (Coveney et al., 2022a). IFT88 deletion in cartilage altered chondrocyte differentiation and mineralization by reducing ciliation in the growth plate. These effects were mostly limited to the peripheral tibial regions under the knee’s load-bearing chambers (Figure 7C). AggrecanCreER T2; Ift88 fl/fl mice had fewer and lower density bone bridges than controls. This decrease in bridging was notably noticeable on the medial side of the leg (Figure 7D). The authors argue that ciliary IFT88 protects coordinated ossification of the growth plate from an disruptive heterogeneity of physiological mechanical stimuli during this critical stage in adolescent skeletal maturation.

Primary cilia are essential for the formation of mammalian tissues. Although primary cilia are important for chondrocyte function, their specific roles in postnatal articular cartilage morphogenesis are unknown. Rux et al. used a mouse conditional loss-of-function method (Ift88-flox) targeting joint-lineage progenitors (Gdf5Cre) to investigate the mechanisms. They discovered that tidemark patterning and hedgehog signaling were substantially disturbed, and that specificity was demonstrated based on regional load-bearing functions of articular cartilage (Rux et al., 2022).

#### 4.1.2 Integrins

Integrins consisting of α and β subunits, translocate across the cell membrane. Numerous experiments have established that chondrocytes express a number of integrins, including integrins α5β1, αVβ3, αVβ5, α6β1, α1β1, α2β1, α10β1, and α3β1 (Loeser et al., 2000; Lahiji et al., 2004; Shattil et al., 2010; Tian et al., 2015). The protein complex is given the name “integrin” to signify its function as an integral complex involved in the transmembrane interaction between the cytoskeleton and ECM (Tamkun et al., 1986). Integrins are well-known as ECM receptors and cell adhesion molecules. They are also thought to affect intracellular signaling pathways physically and chemically as mechanoreceptors. Through integrin-mediated adhesion, cells detect and react to the elastic properties of ECM. Integrins are a class of well-known mechanosensors in cells that alternate between the inactive, bound, and dissociated states based on the various forces acting on them (Xu et al., 2014). When osteoarthritic chondrocytes and normal chondrocytes are mechanically stimulated, there are obvious differences in the cellular responses. These differences could be connected to variations in integrin expression and function [61].

**FIGURE 8:**
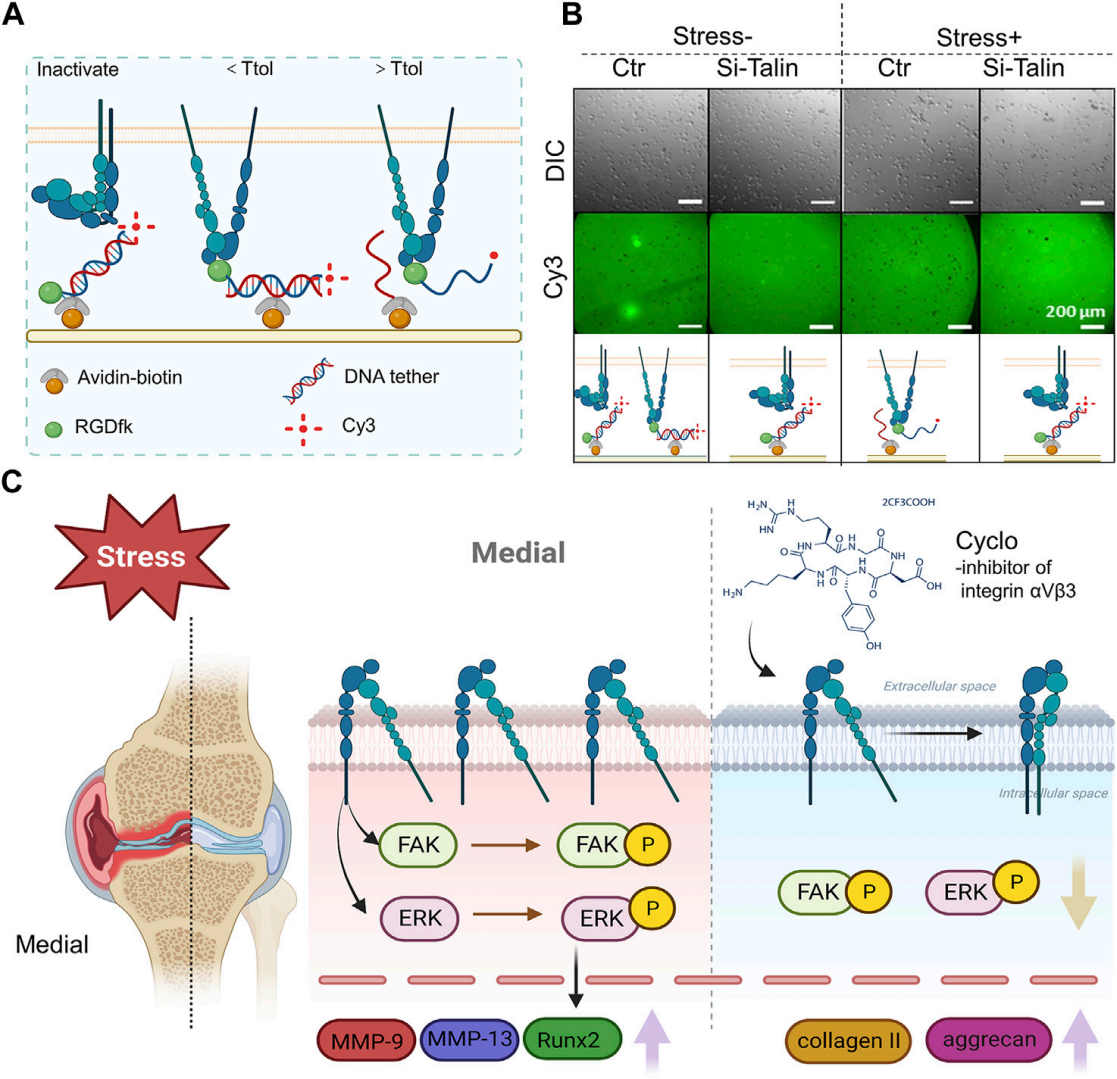
The role of integrin αVβ3 signaling in the development of osteoarthritis brought on by high levels of mechanical stress **(A)** An avidin-biotin linker is used to immobilize a dsDNA tether on the PEG surface. The tension tolerance (Ttol) of the dsDNA is determined by the position of the biotin. The cell-expressed integrins attach to the RGDfk coupled with dsDNA at one end. If the tension applied by the cell through the integrin-RGD link is greater than its Ttol, the dsDNA ruptures. If the tension applied by the cell through the integrin-RGD connection is less than its Ttol, the Cy3 fluorescence signals and cell attachment on the PEG surface are maintained (figure created using BioRender.com). **(B)** Direct imaging of RGDfK-dsDNA-Cy3 removal on the PEG surface (bottom row) and representative differential interference contrast images of SV40 cells on the PEG surface (top row). The cells were subjected to a prechallenge with or without fluid shear stress (Zhen et al., 2021). **(C)** Integrin αVβ3 is overexpressed in the area of the OA knee joint that bears weight. In rat chondrocytes, phosphorylation of FAK and ERK encouraged the production of inflammatory and degradative mediators associated with osteoarthritis. Upregulation of collagen II and aggrecan expression was caused by integrin αVβ3 inhibition (Song et al., 2023) (figure created using BioRender.com).

The traction force between the αV integrin and its ligand is increased by mechanical force, suggesting that the αV integrin-RGD link may survive molecular tensions of up to 54 pN in chondrocytes under mechanical stress. When αV integrin bonds with its RGD-containing ligands, such as latent TGF, in chondrocytes under mechanical stress or not, Zhen et al. used a double-stranded deoxyribonucleic acid (dsDNA) tether as a tension gauge to test the tolerance to tension (Figures 8A,B) (Zhen et al., 2021). Mechanical stimulation of healthy chondrocytes resulted in enhanced GAG production, which was prevented by antibodies to α5 and αVβ5 integrins, as well as CD47 (Holledge et al., 2008). These findings show that αVβ5 integrin plays a significant roles in influencing chondrocytes responses to biomechanical stimuli. The subunits of integrins β1 and 3 are both necessary for osteocyte mechanotransduction. In osteocytes, inhibition of these integrin subunits resulted in poorer responses to fluid shear stress (Geoghegan et al., 2019). Integrin α1β1 has been identified within the integrin family as a critical participant in transducing hypo-osmotic stress. It has been demonstrated that deleting the integrin α1 subunit inhibits chondrocytes’ *ex vivo* and *in vitro* production of Ca^2+^ transients in response to hypo-osmotic stress (Jablonski et al., 2014).

The constant external stimulation that chondrocytes experience controls remodeling. The maintenance of chondrocyte homeostasis requires an ideal degree of mechanical stimulation, but excessive mechanical stress results in the production of inflammatory cytokines and proteases such as matrix metalloproteinases (MMPs). Using an integrin receptor antagonist (cilengitide), Hirose et al. investigated the relation between integrins (αVβ3 and αVβ5) and the production of inflammatory markers in chondrocytes under mechanical loading. Interleukin-1 (IL-1), tumor necrosis factor (TNF), matrix metalloproteinase-3 (MMP-3), and MMP-13 gene expression that was increased by severe mechanical stress was inhibited by cilengitide (Hirose et al., 2020). Through the activation of the TGF-1/CCN2/integrin-5 pathway, high mechanical stress also causes chondrocyte fibrosis, and halting the expression of TGF-1, CCN2, or integrin-5 can reduce the fibrous development (Huang Y. Z. et al., 2021).

Recent study evidence indicates that excessive mechanical stress (eMS) is an important contributor in the development of OA. In a rat instability of the medial meniscus model, histologic and proteomic analysis of osteoarthritic cartilage revealed increased expression of integrin αVβ3 as well as more severe cartilage degeneration in the medial weight-bearing region (Figure 8C) (Song et al., 2023).

#### 4.1.3 Ion channels

Based on their gating methods, ion channels can be divided into a number of groups, including voltage-gated, ligand-gated, and mechanically-gated. The chondrocyte has different types of channels, but mechanically gated ion channels are particularly intriguing since they can trigger rapid mechanosensory signal transduction. Mechanosensitive ion channels control ECM generation and matrix protein synthesis in articular chondrocytes. This mechanism involves intracellular cation efflux, extracellular cation influx, and mobilization of Ca^2+^ as a result of large Ca^2+^ release from storage (Zhang K. et al., 2021). The initial responses of chondrocyte mechanotransduction involve changes in mitochondrial activity and calcium influx, which take place in seconds to minutes (Delco and Bonassar, 2021). The levels of calcium, an universal messenger, regulate a number of critical cellular functions, such as exocytosis, apoptosis, motility, gene transcription, and differentiation (Uzieliene et al., 2018).

TRPV4 and Piezo 1/2 are arguably the most significant calcium channels. TRPV4 was initially identified in articular chondrocytes as an osmotically sensitive Ca^2+^ ion channel, and it was later demonstrated to be sensitive to physiological dynamic compression (Delco and Bonassar, 2021). Additionally, it was found that the mechanically gated Ca^2+^ channels Piezo 1/2 in articular chondrocytes respond to high (supraphysiologic) strain and are in charge of mechanically inducing chondrocyte death (Zhang M. et al., 2022). The potential roles of TRPV4, Piezo1/2 in translating different magnitudes of repetitive mechanical stimuli in chondrocytes were identified in a study. To investigate this, TRPV4, and Piezo1/2 specific siRNAs were transfected into cultured primary chondrocytes to inhibit the expression of TRPV4, Piezo1, or Piezo2, respectively. These cells were then referred to as TRPV4-KD (knock down), Piezo1-KD, or Piezo2-KD cells. Stretch-evoked Ca^2+^ fluctuations were markedly reduced in TRPV4-KD, Piezo1-KD, or Piezo2-KD cells as compared to control siRNA-treated cells, demonstrating the necessity of these channels for Ca^2+^ signaling in chondrocytes generated by stretch stimulation. Notably, these channels responded differently to the calcium oscillation brought on by different stretch stimulation intensities. More specifically, Piezo2-mediated Ca^2+^ signaling was critical for chondrocyte response to damaging levels of strain (18% of strain), whereas TRPV4-mediated Ca2+ signaling was critical for chondrocyte response to normal levels of strain (3% and 8% of strain) (Du et al., 2020). The idea of therapeutically targeting Piezo2-mediated mechanotransduction for the therapy of cartilage disease is prompted by the results, which serve as a foundation for further research into mechanotransduction in cartilage.

GsMTx4, a PIEZO-blocking peptide, and Piezo1/2-specific siRNA blocked mechanically induced Ca^2+^ transients produced by atomic force microscopy in primary articular chondrocytes (Lee et al., 2014), proposing a potential therapeutic approach to attenuate Piezo-mediated cartilage mechanotransduction of damaging stresses to reduce cartilage injury and posttraumatic osteoarthritis. Interleukin-1 (IL-1) was discovered to upregulate Piezo1 in porcine chondrocytes. The enhanced Piezo1 function caused excess intracellular Ca^2+^ both at rest and in response to mechanical deformation. High resting state Ca^2+^ enhanced mechanically generated deformation microtrauma via rarefying the F-actin cytoskeleton (Lee et al., 2021).

Osteoarthritis and joint arthropathy are linked to the loss of TRPV4 function, which is a Ca^2+^-permeable osmomechano-TRP channel that is extensively expressed in articular chondrocytes. The acute, mechanically mediated regulation of proanabolic and anticatabolic genes has been demonstrated to be prevented by TRPV4 inhibition during dynamic loading. It has also been shown to impede the loading-induced augmentation of matrix accumulation and mechanical characteristics. Additionally, in the absence of mechanical loading, pharmacological stimulation of TRPV4 by the agonist GSK1016790A promoted anabolic and inhibited catabolic gene expression, potently increased matrix production, and improved the mechanical characteristics of the construct (O'Conor et al., 2014). These results lend credence to the idea that mechanical cues that maintain joint health and cartilage extracellular matrix preservation are primarily transmitted by TRPV4-mediated Ca^2+^ signaling.

A study shows that loss of TRPV4-mediated cartilage mechanotransduction in adulthood lessens the severity of aging-associated OA by using tissue-specific, inducible TRPV4 gene-targeted mice. These findings point to a unique disease-modifying strategy for the treatment of OA associated with aging by therapeutically targeting the TRPV4-mediated mechanotransduction pathway (O’Conor et al., 2016). However, blocking TRPV4-mediated calcium ion transmission was insufficient to stop the progression of OA on its own. Ion channels belonging to the Piezo family have recently been found to regulate chondrocyte damage response and cell death, as well as giving chondrocytes mechanosensitivity to high stresses (Lee et al., 2014). Therefore, multimodal therapy strategies may be required.

#### 4.1.4 Growth factors

The growth factor is produced as the inactive latent complex that cannot attach to membrane receptors cause a cellular biological response. Recent research has shown that mechanical stresses may activate dormant growth factors. Growth factors are trapped within the pericellular matrix. Increased bioavailability of these upon mechanical stimulation leads to chondrocyte activation. The PCM and territorial matrix’s composition and structure affect the bioavailability of sequestered growth factors like fibroblast growth factor (FGF) (Vincent et al., 2007; Vincent, 2011), transforming growth factor-β (TGFβ) (Albro et al., 2012; Li et al., 2012) and insulin-like growth factor (IGF) (Martin et al., 2002). ECM-sequestered factors are released during deformation or destruction to interact with cell membrane receptors, thus activating downstream intracellular signaling cascades.

The pericellular matrix of articular chondrocytes contains a highly abundant growth factor called FGF2. The location of FGF-2 storage in articular cartilage, the proteoglycan to which it was linked, and its function in chondrocyte mechanotransduction were all uncovered by a study. In articular cartilage, heparan sulphate proteoglycan traps FGF-2. In the type VI collagen-rich pericellular matrix of pig and human articular cartilage, perlecan and FGF-2 co-localize. Chondrocytes enclosed with alginate had the capacity to build up pericellular perlecan and FGF-2 in culture and to activate ERK in a FGF-dependent manner when loaded (Vincent et al., 2007). Studies exploring the function of FGF-2 have shown inconsistent results. The two main articular cartilage FGF receptors, FGFR1 and FGFR3, may have changed in balance, which could explain variations in responses to FGF-2. The majority of FGF2’s catabolic and anti-anabolic actions are mediated by FGFR1, whereas the positive effects are handled by FGFR3 (Vincent, 2011).

It has been discovered that delayed compressive stress induces endogenous TGF-1 gene transcription, protein expression, and subsequent activation even when exogenous TGF-1 stimulation is stopped (Li et al., 2012). Shearing synovial fluid may have extra metabolic effects on diarthrodial joints. It was also shown that TGF-β could be activated in cell-free scaffolds, proving that mechanical stress alone is, at least in part, responsible for the observed activation (Albro et al., 2012). Further, an investigation revealed the functions of TGF-β/SMAD and integrin signaling, indicating cross-talk between these two signaling pathways in controlling the development of compression-driven hypertrophy (Zhang et al., 2015).

A number of lines of research indicate that the anabolic cytokine IGF-I is important for maintaining articular cartilage and may even be involved in cartilage repair. IGF-I increases chondrocyte synthesis of matrix macromolecules. The high co-localization of the pure IGFBP-3 and fibronectintwo in the cartilage matrix and the direct binding between them provide evidence in favor of the theory that these two proteins work together to control local IGF-I levels (Martin et al., 2002).

### 4.2 Downstream pathways

**FIGURE 9:**
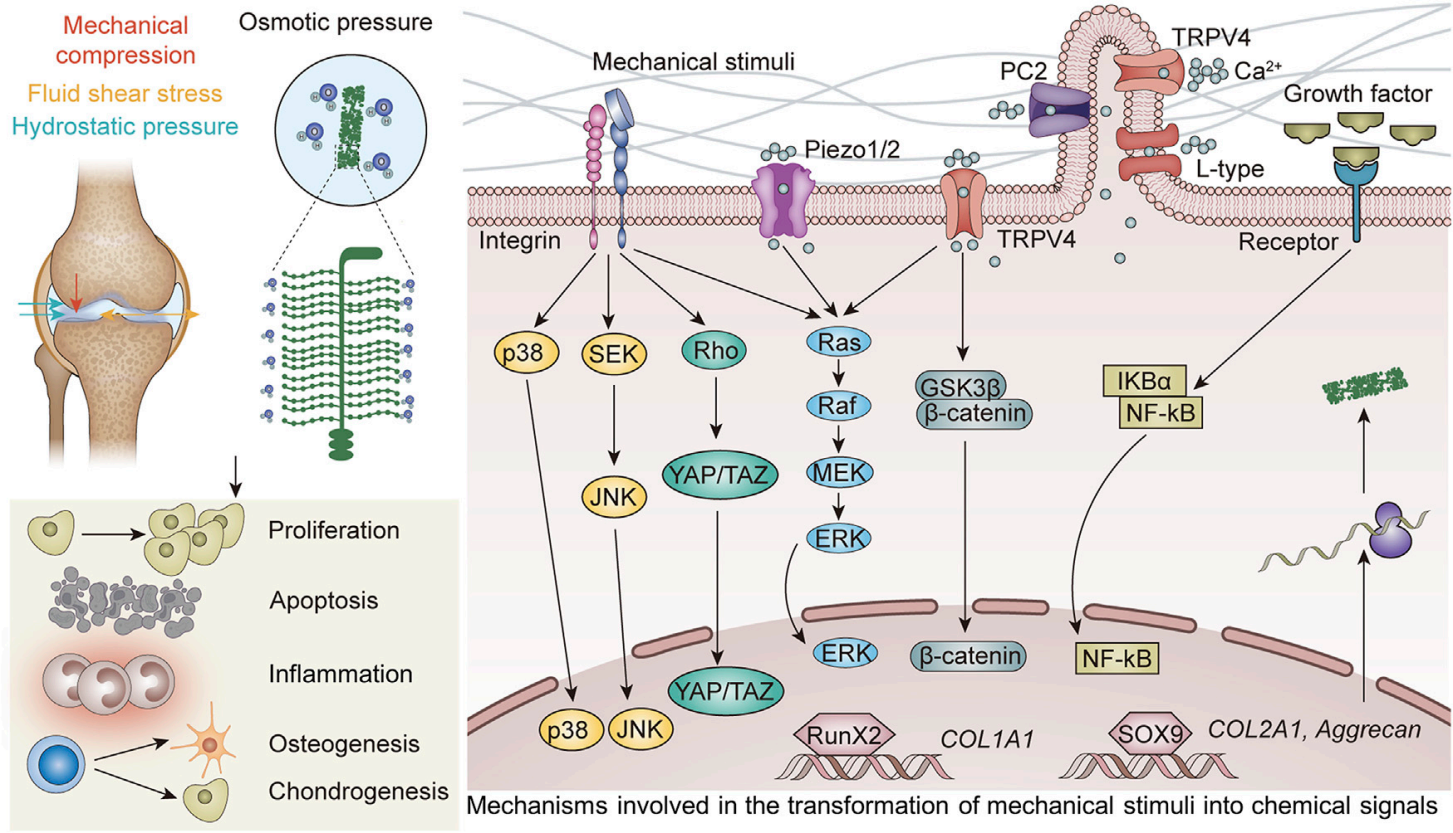
Mechanosensors transform mechanical stimuli into chemical signals, and the genes linked to anabolic or catabolic chondrocyte processes are expressed in a context-dependent manner as a result of the activation of downstream pathways.

The expression of genes linked to either anabolic or catabolic chondrocyte processes occurs from the activation of certain downstream pathways (Figure 9; Table 1).

**TABLE 1:** Studies from the last 5 years focusing on mechanotransduction mechanisms.

Mechanical stimuli	Magnitude	Cell type	Downstream pathway	Most important contributions	Ref
Dynamic compression	15 V and 2 Hz (sinusoidal waveform)	Chondrocyte (rat)	Integrin αVβ3/FAK/ERK	Expression of inflammatory and degradative mediators as MMP-13, MMP-9, ADAMTS-5, and Runx2 was upregulated	Song et al. (2023)
Dynamic compression	20 kPa (2 s on, 1 s off)	Chondrocyte (mouse)	AT1R/JNK	Col X and Runx2 mRNA expression was increased, which promoted hypertrophic differentiation	Nakamura et al. (2018)
	20 kPa (static offset)				
Elongation strain	20%, 24 h	Chondrocyte (mouse)	FBXW7/MKK7/JNK	Increased *p16INK4A*, *p21*, and *COlX* and downregulated *COLIIA1* and *ACAN*, caused chondrocyte senescence and accelerated cartilage catabolism, exacerbating OA.	Zhang et al. (2022a)
Dynamic compression	5%, 0.2 Hz	Chondrocyte (human)	p-ERK/p38 kinases	ACAN and COLII levels that are increased show that dynamic loading with 5% strain has chondrosupportive effects	Xie et al. (2021)
			RhoA/YAP		
Cyclic tensile strain	20%, 6 cycles/min	Chondrocyte (OA patient)	GPER/YAP	Actin polymerization and the RhoA/LIMK/cofilin pathway were inhibited by mechanical stress	Sun et al. (2021)
Cyclic mechanical tension	10%, 0.5 Hz	Chondrocyte (patient with lumbar disease)	Nuclear localization of p-YAP	Mechanical stimuli enhanced the activity of the ACAN or COL2A1 promoter and postponed the degradation of endplate cartilage	Ding et al. (2022)
Biomaterial stiffness	Slower-degrading, stiff	BM-MSCs (human)	Nuclear localization of p-YAP	Mechanical stimuli promoted the development of cells into hypertrophic chondrocytes and showed greater levels of Runx2, a typical hypertrophy marker	Lee et al. (2020)
Biomaterial stiffness	NX-MC(0.34 ± 0.11 kPa)	MSCs (human)	Nuclear localization of p-YAP	*COL1A1* and *RUNX2* expression was increased, along with the expression of osteogenic markers and mineralization	Zhou et al. (2021)
	MC(3.90 ± 0.36 kPa)				
Dynamic compression	25% cyclic (10 min)	Chondrocyte (human)	Wnt/β-catenin	Decreasing the ECM content and suppressed the upregulation of SOX9	Praxenthaler et al. (2018)
	10% static (10 min)				
	9 times				
Hydrostatic pressure	10 Mpa	Chondrocyte (OA patient)	Wnt/β-catenin	The expression of Col2a1, proteoglycan, and aggrecan was shown to be downregulated, whereas MMPs and ADAMTS were upregulated. Apoptosis signaling was also enhanced	Cheleschi et al. (2020)
	3 h				
Hydrostatic pressure	0.9 Mpa	Meniscus fibrochondrocyte (female)	Wnt/β-catenin	Upregulated the average COL2A1 expression level 215.9-fold was shown in the CHP group for the female cohort	Ma et al. (2022)
	1 Hz				
Mechanical stretch	20%, 1 Hz	Osteoblast and Chondrocyte (rat)	Wnt/β-catenin	Collagen 1a and alkaline phosphatase reduced, and MMP 13, IL-6, and PGE2 increased in osteoblasts. In chondrocytes, there was a decrease in collagen 2a and an increase in MMP-13, the disintegrin, and metalloproteinase 5 with thrombospondin-like motifs	Song et al. (2021)
Hydrostatic pressure	20 MPa	Chondrocyte (mouse)	Gremlin-1/NF-κB	Sox9 and the cartilage matrix genes Col2a1 and Acan were expressed less, whereas catabolic marker genes including MMP-13 and ADAMTS-5 were expressed more	Chang et al. (2019)
	1 Hz				
Mechanical stretch	20%	Nucleus pulposus cell (human)	Ca2+/NF-κB	Promoted NLRP3 inflammasome assembly and upregulated caspase-1 activation and IL-1β production	Sun et al. (2020)
	6 cycles/min				
Dynamic compression	25% cyclic (10 min)	BM-MSC (human)	Nuclear localization of NF-κB	The expression of COX2 and BMP2 was higher, PGE2 synthesis was more than 100 times higher, and SOX9 stimulation was less than the control group	Lückgen et al. (2022)
	10% static (10 min)				
	9 times				
Biomaterial stiffness	PC (4.3 MPa)	BM-MSCs (rat)	Blocking the NF-kappa B signaling pathway	Lowered the expression of Ltb, Traf1, Vcam1, and Mmp13 and elevated that of cartilage-specific genes Col2a1, Acan, and Sox9 in PC.	Jiang et al. (2018)
	P (6.8 MPa)				

#### 4.2.1 MAPK pathway

A number of cell signaling pathways involved in cellular proliferation, the production of the extracellular matrix (ECM), cell survival, and the mediation of pain are controlled by the mitogen-activated protein kinase (MAPK). The MAPK pathway, which is involved in mechanotransduction and is controlled by mechanosensory stimuli, is essential for chondrocyte differentiation. TRPV4, p38, and primary cilia are all necessary to activate ERK.

According to one study, *ex vivo* cartilage compression increases the activation of the JNK pathway enzymes SEK1, p38 MAPK, and ERK1/2. This work also indicates unique temporal patterns of MAPK signaling in response to mechanical stress and that mechanical compression alone can activate MAPK signaling in healthy cartilage (Fanning et al., 2003). Various clinical studies have found that the MAPK pathway plays an essential role in the cartilage development (Xiang et al., 2019), cartilage catabolism (Zhang H. et al., 2022), expression of inflammation-related factors (Yanoshita et al., 2018), and enhancement of chondrogenesis (Xie et al., 2021).

Magnitude-dependent effects of mechanical stress on chondrocyte survival, phenotypic, and proliferation have been observed. The expression of autophagy and mechanical stress-regulated ERK/mTOR signaling in chondrocytes depend on the structural integrity of primary cilia (Xiang et al., 2019). In mice articular cartilage and cultured chondrocytes, mechanical overloading speeds up senescence. Mechanical overloading reduces the transcription of the F-box and WD repeat domain containing 7 (FBXW7) mRNA and the MKK7 degradation caused by FBXW7, which in turn stimulates JNK signaling. As evidenced by the overexpression of p16INK4A, p21, and Colx and the downregulation of Col2a1 and ACAN, FBXW7 deletion in chondrocytes has been found to cause chondrocyte senescence and accelerate cartilage catabolism in mice, which led to the worsening of OA (Zhang H. et al., 2022).

The non-receptor tyrosine kinase focal adhesion kinase (FAK) is connected to numerous signaling proteins. The phosphorylation of FAK, p-38, ERK, and JNK was triggered by cyclic tensile strain, which also elevated the expression of the genes encoding COX-2, IL-1, and TNF-α. Through MAPK pathways, FAK seems to control inflammation in chondrocytes exposed to cyclic tensile strain (Yanoshita et al., 2018). Dynamic compression raised the compressive moduli of manufactured cartilage tissues and encouraged the formation of cartilage matrix. ACAN and COL2 levels were increased in constructs cultivated under dynamic loading conditions, confirming the function of dynamic loading with 5% strain as a chondro-supportive agent (Xie et al., 2021).

Under high-strain activation, downstream signaling molecules MAPK/ERK1/2 and MAPK/ERK5 cause late excessive death of chondrocytes through the action of Piezo1 channels. Through the traditional MAPK/ERK1/2 signaling pathway, Piezo1 plays a crucial role in the apoptosis of the human chondrocytes (Li et al., 2016). Following apoptosis, the chondrocyte stimulates the joint’s surroundings and releases a lot of oxygen free radicals and inflammatory mediators (including IL-1, TNF, and PE) that harm the newly formed cartilage tissue and blood vessels (Liu et al., 2022). After pre-incubation at 380 mOsmol, it was discovered that exposure to hyperosmotic conditions (550 mOsmol) initially reduced the rate of 35S-sulphate incorporation. However, after 24 h of culture, rates bounced back and even exceeded their pre-exposure values. This reaction was eliminated by MAP kinase inhibitors, which suggests that they are involved in the adaption mechanism (Hopewell and Urban, 2003). Therefore, it is believed that a number of mechanosensory stimuli have the MAPKERK pathway as a downstream target.

#### 4.2.2 YAP-TAZ pathway

Chondrogenesis, chondrocyte maturation, and hypertrophy are all controlled by the transcriptional cofactors Yes-associated protein (YAP) and transcriptional coactivator with PDZ-binding motif (TAZ), which together make up a crucial mechanosignaling complex. The roles of YAP and TAZ as nuclear relays of mechanical stimuli exerted by ECM stiffness and cell shape were identified (Dupont et al., 2011).

The transcriptional cofactors YAP and TAZ form an important mechanosignaling complex and are involved in regulating mechanical stress-mediated apoptosis of chondrocytes (Sun et al., 2021), the degeneration of chondrocytes (Ding et al., 2022), chondrocyte hypertrophy (Lee et al., 2020) and cartilage degradation (Gong et al., 2019).

The expression of the G protein coupled estrogen receptor (GPER) is negatively linked with the pathophysiology of OA cartilage degradation. By encouraging the expression of YAP and ARHGAP29 as well as YAP nuclear localization, GPER suppresses Piezo1 and the mechanical-stress-mediated RhoA/LIMK/cofilin pathway as well as actin polymerization (Sun et al., 2021).

In mechanical-tension-mediated degenerative discs, YAP1 is a crucial regulator. In degenerative human endplate cartilage tissue, YAP1 expression has been shown to be dramatically reduced with higher mechanical stimulation intensity and duration. Additionally, it has been demonstrated in the cartilage endplate tissue *in vitro* that increasing the expression level of YAP1 can postpone the degeneration of endplate cartilage (Ding et al., 2022).

Although biomaterial design solutions for repairing injured articular cartilage have advanced significantly, preventing stem-cell-derived chondrocyte hypertrophy and the subsequent creation of inferior tissue remains a significant difficulty (Lee et al., 2020).

Furthermore, it has been discovered that OA cartilage tissue expresses higher levels of YAP1, and that YAP1 that is overexpressed interacts with Beclin 1 to advance OA. In a mouse model of mechano-induced OA, researchers attempted to employ siRNA to block YAP1, and they discovered that doing so avoided cartilage breakdown and improved OA development (Gong et al., 2019).

Both in MSCs and chondrocytes, the amount of fluid flow stress controls the expression of YAP. An increase in the stimulation magnitude enhanced the expression of YAP, increasing osteogenesis and initiating dedifferentiation for chondrocytes (Zhong et al., 2013). According to Karystinou et al., YAP is a negative regulator of MSCs’ chondrogenic development. Through the derepression of chondrogenic signaling, YAP must be downregulated for chondrogenesis (Karystinou et al., 2015). A promising future in rheumatology involves therapeutic targeting of YAP to promote cartilage repair and avoid subsequent osteoarthritis. These findings demonstrate the importance of YAP/TAZ as effectors that may transmit mechanical stress into the nucleus and support healthy chondrocyte formation, maturation, and homeostasis.

#### 4.2.3 Wnt pathway

Wnt signaling has established roles in the expression of hypertrophic markers (Lee et al., 2020), in decreasing the ECM content of cartilage (Praxenthaler et al., 2018), chondrocyte metabolism and oxidative stress (Cheleschi et al., 2020), pro-chondrogenic effects (Ma et al., 2022), and osteoblast osteogenic differentiation (Song et al., 2021). The Wnt signaling pathways that support increased expression of hypertrophic markers in cartilage can affect how transplanted articular or hypertrophic phenotypes behave. Encapsulated hMSCs were pretreated with Wnt inhibitors for 21 days in order to stop them from developing along hypertrophic pathways. Wnt inhibitor supplementation reduced the expression of indicators linked to hypertrophic chondrogenesis in comparison to controls without the addition of inhibitors, which showed an increase in the expression of hypertrophic markers (Lee et al., 2020).

WNT/-catenin and pSmad1/5/9 levels decreased as cartilage’s ECM content rose. In mature constructs, the Wnt agonist CHIR reduced load-induced SOX9-and GAG activation by increasing -catenin levels. IWP-2, a WNT antagonist, on the other hand, had the ability to lessen the GAG-suppression caused by load in developing constructs. In conclusion, a stronger anabolic response of chondrocytes to physiological loading was enabled by either ECM accumulation-associated or chemically induced silencing of WNT-levels (Praxenthaler et al., 2018).

According to one study, hydrostatic pressure (HP) controls chondrocyte metabolism and oxidative stress via the Wnt/-catenin pathway in part by silencing certain miRNAs. Low cyclical HP substantially lowered the amounts of apoptosis, MMP-13, ADAMTS5, miRNA, superoxide anion generation, and mRNA for antioxidant enzymes. On the other hand, Col2a1 and BCL2 genes showed greater expression. The application of continuous static HP produced opposite consequences. Finally, miRNA silencing improved low HP and blocked effects of ongoing HP (Cheleschi et al., 2020). In a different study, mechanical loading by cyclic hydrostatic pressure (CHP) had a pro-chondrogenic impact, whereas mechanical unloading by simulated microgravity (SMG) produced OA-like gene expression in manufactured cartilage. Each sex group displayed a unique gene profile. For instance, the NOTUM gene, which is a part of the Wnt signaling pathway, was considerably elevated in the CHP for the female cohort by 6.7-fold but only by 1.8-fold in the CHP for the male cohort. However, SMG had a negligible impact on NOTUM regulation, and it revealed the opposite direction between male and female cohorts (Ma et al., 2022).

In addition to inhibiting osteoblast osteogenic development, severe mechanical stretching of osteoblasts also caused chondrocyte catabolism and apoptosis. This was accomplished by the Wnt/catenin signaling pathway (Song et al., 2021).

Suppressing canonical Wnt signaling may enhance the chondrogenesis of MSCs and attenuate the progression of OA. In comparison to control hydrogels, encapsulating hMSCs in these self-assembled N-cadherin mimic peptide hydrogels resulted in increased expression of chondrogenic marker genes and deposition of extracellular matrix specific to cartilage that is rich in proteoglycan and Type II collagen. Western blot assessment revealed a substantially reduced level of -catenin and a significantly higher expression of active glycogen synthase kinase-3 (GSK-3), which phosphorylates catenin and promotes ubiquitin-mediated destruction. In N-cadherin mimicking peptide hydrogels, immunofluorescence labeling showed much less nucleus localization of catenin. According to the results, N-cadherin peptide hydrogels increase the chondrogenesis of hMSCs by increasing catenin nuclear translocation and the transcriptional activity of the catenin/LEF-1/TCF complex and suppressing canonical Wnt signaling in hMSCs (Li et al., 2017).

GSK3β is also a downstream protein of TRPV4. In order to regulate GSK3 activation, normal chondrocytes’ intracellular calcium levels, which are controlled by TRPV4 ion channels, fluctuate in response to the viscoelasticity of the ECM. Additionally, osteoarthritic chondrocytes’ TRPV4-GSK3 molecular axis has been damaged, which prevents OA patients’ cells from sensing and reacting to the changed viscoelasticity of the surrounding matrix (Agarwal et al., 2021).

#### 4.2.4 NF-κB pathway

The development of osteoarthritis is heavily influenced by the excessive forces that the articular cartilage is exposed to. Under cyclic strain or hydrostatic pressure loading, Gremlin-1 is found to be a mechanical loading-inducible factor in chondrocytes and is strongly expressed in the middle and deep layers of cartilage. Nuclear factor-B signaling is activated by Gremlin-1, which causes the production of catabolic enzymes (Chang et al., 2019). Osteoarthritis progression is slowed in mice by intra-articular infusion of Gremlin-1 antibody or chondrocyte-specific Gremlin-1 deletion, but this progression is sped up by intra-articular administration of recombinant Gremlin-1 (Chang et al., 2019). These findings point to NF-B’s pivotal function in mechanoinflammation as OA progresses, but perhaps more significantly, they point to several intriguing treatment targets.

In addition to being triggered by cytokine signaling, the physical pressures within chondrocytes can also regulate NF-B. NF-κB activation can activate inflammation (Sun et al., 2020), mimic the negative loading effects (Lückgen et al., 2022), and lead to the induction of catabolic enzymes (Chang et al., 2019). A study found evidence connecting the development of NLRP3 inflammasome with Piezo1-mediated inflammation in nucleus pulposus cells. A unique pathogenic mechanism driving the development of intervertebral disc degeneration is the activation of NLRP3 inflammasome in nucleus pulposus cells via Piezo1 through the Ca^2+^/NF-B pathway (Sun et al., 2020). While catabolic NF-B signaling prevents load-induced deleterious effects on ECM synthesis in MSC-derived neocartilage, NF-B activation mimics negative loading effects and increases PGE2 production (Lückgen et al., 2022). The mechanical characteristic of neocartilage generated from mesenchymal stromal cells is increased by the NF-B suppression.

The new PCL-PTHF urethane electrospun nanofibers with collagen I from calf skin were found to be more effective at inducing chondrogenic differentiation *in vitro* and cartilage regeneration *in vivo* than the stiffer P PCL-PTHF urethane nanofibers. This was true even in the absence of additional chondrogenesis inducers. The researchers discovered that the PC worked better than P at initiating chondrogenesis by specifically blocking the NF-B signaling pathway to reduce inflammation (Jiang et al., 2018). Another study found that the AMPK/NF-B signaling pathway might be used to regulate the sensitivity of articular cartilage and chondrocytes to the inflammatory response. By promoting AMP-activated protein kinase (AMPK) activation and inhibiting nuclear factor (NF)-B translocation, cyclic tensile strain (CTS) may reduce the chondrocyte damage brought on by IL-1 (Yang et al., 2019).

## 5 Application of mechanical stimuli to chondrocytes

Regenerative cartilage biology involves imitation of *in vivo* cartilage formation and maintenance processes. Current developments in the field of biomaterials engineering center on the use of different alterations and biophysical stimulation of scaffolds to create implants that support cartilage regeneration.

### 5.1 Biomaterials for cartilage engineering mimic the mechanical properties of natural cartilage

Using cartilage tissue engineering (TE) for healing sick or damaged tissue is a promising new method. Biomaterial engineering aims to fabricate implantable biocompatible scaffolds that accelerate tissue regeneration (Przekora, 2019). It should be noted that the incapacity of biomaterials to accurately mimic the mechanical properties and resist the load of the original cartilage must be overcome in order for them to be successfully used for cartilage TE. The anisotropy of the tissue, which enables the liquid phase of the cartilage to migrate across the solid tissue during loading, is crucial to the complicated mechanical properties of articular cartilage. Body tissues’ mechanical qualities decrease in various disease conditions, making them more prone to additional material failure. The ultimate objective is to create materials with mechanical resistance that produce the proper form of cartilage (Dieterle et al., 2021).

Scaffolds imitating the natural mechanical environment for chondrocyte growth have been made from a variety of organic and synthetic materials. In this context, 3D woven fiber scaffolds were used to imitate the mechanical characteristics of the native cartilage. 3D woven poly (epsilon-caprolactone) (PCL) scaffolds seeded with MSCs in MatrigelTM were shown to have aggregate and Young’s moduli that were relatively similar to that of healthy articular cartilage (0.1–2.0 MPa and 0.4–0.8 MPa, respectively) (Valonen et al., 2010). Similar to this, biomechanical testing revealed that fiber-reinforced PCL-based constructs had initial compressive and shear properties that were comparable to those of native cartilage. These constructs also maintained these properties over the course of the culture period while promoting the synthesis of a collagen-rich ECM (Moutos and Guilak, 2010).

#### 5.1.1 Natural materials scaffold

Autologous chondrocyte implantation (ACI) on a collagen type I/III scaffold was investigated by Nixon and coworkers, and it appeared to promote cartilage regeneration in a critical-sized lesion in the equine model over the course of 6 months (Nixon et al., 2015). The chondro-inductive effects of 3D collagen and hyaluronic acid hydrogels—self-assembled collagen hydrogel (Col), self-assembled collagen hydrogel cross-linked with genipin (Cgp), and methacrylated hyaluronic acid hydrogel (HA)—on the encapsulated BMSCs were assessed in a different study. In the subsequent stage, there was not enough room in the hydrogels for cell proliferation due to the extreme shrinkage of Col and Cgp. In contrast, the relatively stable mechanical environment of HA supported the maintenance of the ongoing synthesis of the cartilage matrix in the final stage (Yang et al., 2021).

**FIGURE 10:**
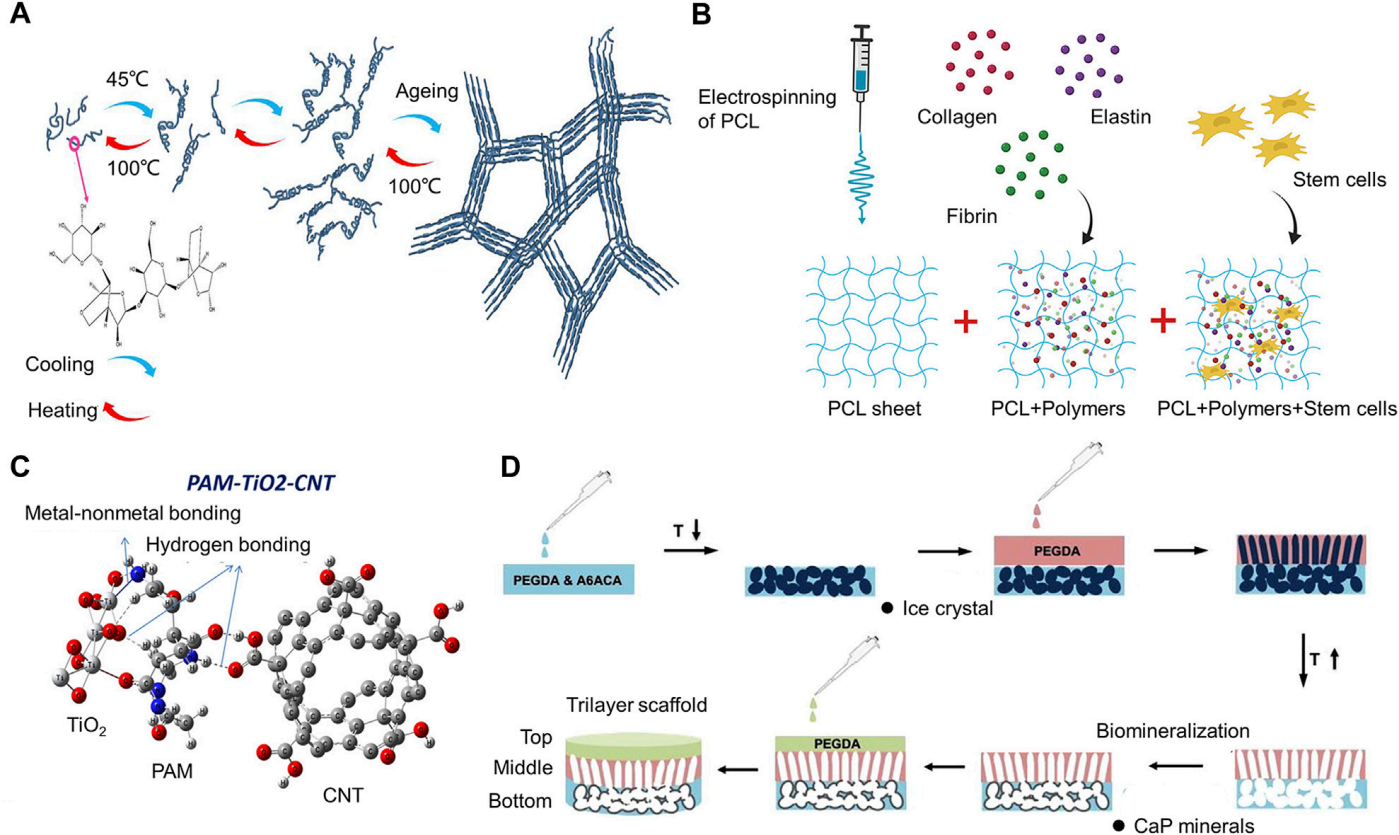
Biomaterials for cartilage engineering mimic the mechanical properties of natural cartilage **(A)** Agarose’s molecular make-up and a diagram of how it gels are shown (Salati et al., 2020). **(B)** Schematic representation of the fabrication of PCL-based composites and addition of human adipose-derived stem cells (Sawadkar et al., 2020). **(C)** The chemical structure of PAM-TiO2-CNT. It has increased mechanical strength and bioactivity due to strong interfacial bonding by TiO2 and CNT with PAM(Awasthi et al., 2021). **(D)** Schematic for stepwise synthesis of the trilayer scaffold (Kang et al., 2018).

Agarose-based biomaterials are crucial in cartilage tissue healing because of their special qualities, including reversible thermogelling behavior and tissue-like mechanical properties (Salati et al., 2020). Induction, gelation, and quasi-equilibrium are the three phases of the agarose gelation process. The initial phase involves the formation of a number of agarose nuclei, which are then grown into networks (Figure 10A).

#### 5.1.2 Synergistic materials scaffold

Scaffolds were made from elastin, collagen, fibrin, and electrospun polycaprolactone (PCL) with varying ratios to overcome the drawbacks of natural and synthetic polymers by combining them to create a synergistic relationship (Figure 10B). As a result, it was possible to modify the physical and biological characteristics of PCL-based composites, in order to create a beneficial connection for a variety of applications in tissue regeneration (Sawadkar et al., 2020).

Polymeric hydrogels hold promise as potential replacement materials for the cartilage that has been injured. As a matrix for autologous chondrocyte implantation, an injectable polyethylene glycol-crosslinked albumin gel (AG) supplemented with hyaluronic acid holds promise as a beneficial implant for cartilage tissue while also exhibiting non-permissive properties for pathological blood vessel formation (Scholz et al., 2010).

#### 5.1.3 Interpenetrating polymer scaffold

The mechanical properties of the original substrate can be increased by incorporating an interpenetrating polymer network into a single polymer network. By transferring this idea from purely synthetic materials to natural-synthetic hybrid systems, the mechanical properties of bulk biological substrates can also be strengthened (Cooper et al., 2016). This procedure improves the osteoarthritic cartilage’s inferior compressive capabilities to those of healthy cartilage. An effective treatment method for the regeneration of posttraumatic or osteoarthritic lesions of the knee, according to a clinical follow-up, is implanting BioSeed-C, which is based on a bioresorbable two-component gel-polymer scaffold (Ossendorf et al., 2007).

Healthy articular cartilage has a complex structure that helps the joint bear external forces, maintain interstitial fluid to reduce strains on its soft tissue, and reduce friction between the cartilages. When the cartilage is under loading, the surrounding matrix reduces the fluid outflow velocity to provide hydrostatic pressure. Early signs of osteoarthritis include the decrease of glycosaminoglycans and the destruction of the collagen network. An innovative polymeric cartilage supplement that recreates the extracellular matrix’s hydrophilic characteristics by forming a charged interpenetrating polymer network (IPN) has been developed (Mäkelä et al., 2018). The hydrophilic ECM is principally strengthened by the IPN in order to recreate the material characteristics of cartilage. The reestablishment of the fluid phase is also impacted by this strengthening of the solid phase.

Hydrogels made of polyacrylamide are frequently used as potential substitutes for cartilage. Their application, however, is severely limited by their low mechanical durability and puncture resistance. The strength of polyacrylamide (PAM) hydrogels was increased by using titanium oxide (TiO2) and carbon nanotubes (CNTs) independently or in combination in a PAM matrix, which was interconnected by bonding between nanoparticles and polymers using a density functional theory (DFT) approach. The PAM-TiO2-CNT hydrogel’s outstanding compressive strength, elastic modulus (>0.43 and 2.340 MPa, respectively), and puncture resistance (estimated using the needle insertion test) are attributed to the synergistic effect and solid interfacial bonding, making it potentially a remarkable nanocomposite hydrogel for cartilage repair applications (Figure 10C) (Awasthi et al., 2021).

#### 5.1.4 Multilayer scaffold

The researchers devised and manufactured a strong bilayer nanocomposite acrylamide-acrylic acid hydrogel reinforced with silica nanoparticles (SNPs). By using only 0.6 wt% SNPs, the mechanical properties revealed a considerable improvement in compressive strength up to 1.4 MPa and a doubled elastic modulus (240 kPa) compared to the non-reinforced hydrogel. With appropriate ratios of monomers and SNPs, samples could be compressed without damage until reaching 85% strain (Mostakhdemin et al., 2021). Bilayer silica-reinforced nanocomposite hydrogels are possible choices for synthetic cartilage due to their mechanical properties.

A biomineralized bottom layer that mimics the calcium phosphate (CaP)-rich bone microenvironment is attached to a cryogel middle layer with an anisotropic pore architecture, and a hydrogel top layer is attached to the trilayer scaffold that is depicted in Figure 10D. The macroporous middle layer and hydrogel top layer were intended to support the production of cartilage tissue, whilst the mineralized bottom layer was intended to support the formation of bones. The transplanted cells continuously differentiated to form cartilage tissue, while endogenous cells were recruited through the mineralized bottom layer to build bone tissue, resulting in the creation of osteochondral tissue (Kang et al., 2018).

#### 5.1.5 Piezoelectric materials scaffold

The capacity of some materials to produce electric signals in response to mechanical stress is known as the piezoelectric effect (Zhang et al., 2018). When cartilage tissue is rebuilding naturally, the extracellular matrix (ECM) creates an electric signal that is reminiscent to that produced by piezoelectric biomaterials. In a physiological environment, the compressive strain on collagen fibers in the ECM causes the dipole moment to reorganize, generating negative charges. As a result, an electrical signal enters the cell membrane, causing voltage-gated calcium channels to open.

According to a study, flexible, 3D fibrous scaffolds made of piezoelectric materials can promote ECM development and human mesenchymal stem cell differentiation under physiological loading circumstances. Piezoelectric scaffolds with a high voltage output encourage osteogenic differentiation while those with a low voltage output or streaming potential encourage chondrogenic differentiation. More considerable differentiation was discovered to be induced by electromechanical stimuli than by mechanical loading alone (Damaraju et al., 2017). Using poly (3-hydroxybutyrate-co-3-hydroxyvalerate) (PHBV) and barium titanate (BaTiO3), a clever piezoelectric nanohybrid was created. Comparatively to the control (pure PHBV) and unpolarized scaffolds, the polarized scaffolds greatly enhance cell adhesion, proliferation, and *COLIIA1* gene expression (Jacob et al., 2019).

#### 5.1.6 Novel materials scaffold

For cartilage tissue engineering, the ideal adaptable scaffolds should evolve dynamically and spatiotemporally in response to the physiological microenvironments at each stage of cartilage repair. ECMs’ mechanical strength changes as a result of growing tissue adjusting to the body’s requirements (Peng et al., 2023b). Therefore, adaptive tissue engineering scaffolds should change their mechanical strength to meet the needs of cells as they grow and mature. In addition, osteochondral tissue is a gradient construct with a seamless transition from cartilage to subchondral bone, involving changes in collagen type and orientation, chondrocyte morphologies, ECM components, and cytokines. To meet the anisotropic properties of osteochondral matrices, bioinspired scaffolds have been created by replicating gradient characteristics in heterogeneous tissues, such as the pores, components, and osteochondrogenesis-inducing substances (Peng et al., 2023a). Bioinspired gradient scaffolds can restore osteochondral defects by modifying the microenvironments of cell development to promote osteochondrogenesis.

Rheumatoid arthritis (RA), is an autoimmune illness brought on by both external (hostile environment and virus invasion) and endogenous (cellular, genic, and neurological issues) components. Today, oral or injectable medications are typically used to treat RA. However, there is an urgent need to create a new method of medication delivery because of the adverse effects such as poor patient compliance, gastrointestinal disturbances, and other toxicity hazards connected with current administrations (Conforti et al., 2021). Hence, transdermal drug delivery systems, represented by microneedles, are becoming popular in the treatments of RA (Zhang et al., 2023). The melittin-loaded MeHA microneedles in the Adjuvant-Induced Arthritis (AIA) animal model prevented the rat’s paw from swelling (Ye et al., 2016). TNF and IL-17 levels also decreased as regulatory CD4+T cells increased, which had a suppressive effect on RA and a protective effect on articular cartilage.

In addition, some new drug-delivery scaffolds can promote angiogenesis and subchondral bone formation, indirectly promoting joint repair (Xu et al., 2023a; Xu et al., 2023b). For the best bone regeneration, designing a multifunctional scaffold with osteogenic and angiogenic capabilities offers promise. Exosomes from human bone mesenchymal stem cells (hBMSCs) were immobilized on porous polymer meshes created by PLGA and Cu-based MOF (PLGA/CuBDC@Exo) in one study to create an innovative scaffold. The manufactured exosome-laden scaffold can deliver a dual cooperative controlled release of bioactive copper ions and exosomes that encourage osteogenesis and angiogenesis, resulting in cell-free bone repair (Xu et al., 2023a). A pro-angiogenic small molecule medication (dimethyloxallyl glycine, or DMOG) was loaded onto an iron-based metal-organic framework (MIL-88) and subsequently embedded into PLGA nanofibrous scaffolds to repair cranial lesions in rats in another study (Xu et al., 2023b). Co-delivery system considerably aided angiogenesis by enhancing endothelial cell migration, tube formation, and osteogenesis by enhancing the production of proteins associated with osteoblasts, according to an *in vitro* study.

If we can combine mechanical stimuli with these state-of-the-art biomaterial scaffolds in cartilage tissue engineering, it is expected to construct grafts that are more consistent with natural cartilage and promote joint repair.

### 5.2 Experimental application to model actual physiological conditions *in vitro*

*In vivo*, the formation and maintenance of articular cartilage depend on mechanical loading, which is a significant component of the articular cartilage environment. Therefore, efforts have been undertaken to incorporate these stresses as additional elements into cartilage engineering by building a number of bioreactors (Uzieliene et al., 2021).

Compression, tensile, and shear deformations combine to build articular motion, thus it is important to identify the precise combination of various mechanical stimuli and create regimens that have the best chondrogenic outcomes. Numerous mechanical stimulation methods have been developed in an effort to mimic the stresses that articular cartilage tissue experiences *in vivo* since it has proven difficult to manufacture cartilaginous tissue with qualities equal to those of native articular cartilage. The sorts of forces, whether they are static or dynamic, constantly applied or intermittently, applied alone or in combination with other types of forces at the same time, and how they are applied all have a significant impact on the outcomes of such systems.

The most often researched mechanical stimulation technique in cartilage tissue engineering is uniaxial static compression on the tissue surface. Designing the matching bioreactor is straightforward and merely calls for basic weights to be applied to structures made of cartilage (Salinas et al., 2018). The dynamic compression bioreactor has a favorable impact on biomechanical moduli and chondrogenic gene expression (Anderson and Johnstone, 2017). In constructs incorporating MSCs, uniaxial compression causes heterogeneous collagen deposition, with surface deposition being the highest. A cartilage-like tissue cannot likely be produced *in vitro* by compression alone as a mechanical signal. Collagen and proteoglycan production were observed to be increased when mechanical loading (5% compression and 5% shear strain amplitudes) was added compared to static (unstimulated) controls (76±8% and 73±5%, respectively) (Waldman et al., 2007).

Human MSCs have been employed in the multifunctional bioreactor. The cell-seeded scaffold was pressed onto a 32 mm ceramic hip ball. The oscillation of the ball about an axis perpendicular to the tissue axis produced the interface shear motion. Along the scaffold’s cylindrical axis, the superimposed compressive strain was applied. Dynamic compression and surface shear were applied for 1 h each day for 7 days after preculture, and this increased hMSC chondrogenesis in comparison to unloaded control samples (Li et al., 2010).

Another study found that the application of shear overlaid atop dynamic compression resulted in much higher levels of chondrogenic gene expression even though no exogenous growth agents were introduced to the culture medium (Schätti et al., 2011).

Cell survival and metachromatic staining were low in TGF-free loaded samples close to the porous compression platen interface, but increased with depth, reaching levels in the deeper part of the hydrogel that were comparable to those of unloaded TGF cultures. According to these findings, low hydrostatic pressure combined with high dynamic strain and fluid flow had a more negative impact on chondrogenesis than high hydrostatic pressure combined with low dynamic strain and fluid flow (Kisiday et al., 2009). The findings might be helpful in guiding the development of enhanced multifunctional bioreactors for chondrogenic differentiation.

Regarding protocols for cartilage engineering that include mechanical stimulation, several aspects should be taken into consideration. The majority of investigations employ bioreactors that were self-designed, which has limited method replication and inconsistent confirmation of the applied forces. Additionally, only certain culture models can be used with mechanical bioreactors. The choice of materials utilized for the scaffolding affects how mechanical stimuli behave. These make data comparisons challenging and ineffective (Grad et al., 2011).

## 6 Conclusion

The development of MSCs into a chondrogenic phenotype and cartilage homeostasis both depend heavily on mechanical stimuli. Advanced bioreactors capable of exerting mechanical compression, fluid shear, hydrostatic pressure, and osmotic pressure or incorporating them enable the *in vivo* environment to be mimicked and enhance the chondrogenic response. Various experiments have proved that only mechanical load in a specific range of magnitude and time is conducive to cartilage regeneration, while maximal and supramaximal mechanical load is detrimental to articular cartilage. Given these advances, our goal is to provide future researchers with the optimal mechanical stimuli parameters (duration, magnitude, frequency, etc.) to promote extracellular matrix production, maintain cell viability, and promote cartilage repair. In addition, we found that different types of mechanical stimuli play different roles, for example, dynamic compression can increase the content of type II collagen and glycosaminoglycan, and fluid shear stress can promote the transport of nutrients. This suggests that we can combine different types of mechanical stimuli and utilize multiple repair mechanisms to build better cartilage tissue-engineered grafts.

However, we also found that the current research has certain limitations. First, there is no unified standard when exploring some parameters of mechanical stimuli. For example, when exploring the effect of dynamic compression, KPa is used as the unit of measurement in some experiments, while the deformation percentage of the structure generated by the compression is used as the unit of measurement in other experiments. Second, most studies have focused on the biological effects of mechanical stimuli, such as collagen content, cell viability, and inflammation. However, less attention is paid to the properties of tissue engineering construct, such as organization, compression, tensile properties, degradability, etc. Therefore, more research is needed to refine the relevant information and help draw a complete conclusion.

Chondrocytes can effectively respond to mechanical stresses either “directly” by detecting PCM deformation through cell-ECM adhesions (primary cilia, integrins) and cell sensors (ion channels), or “indirectly” by causing the release of growth factors and their interaction with cell receptors. Genes linked to anabolic or catabolic chondrocyte processes are expressed in response to context when downstream pathways are activated. Molecular biological mechanisms are elucidated, and moderate or excessive mechanical stimuli conduct biological signals by activating ion channels on the cell membrane, ultimately producing beneficial or adverse biological effects. This suggests that we may be able to use some mechanical receptor agonists or inhibitors to promote beneficial effects and avoid adverse effects, guiding the application of drug therapy in tissue-engineered constructs. Hopefully, deeper comprehension of these mechanisms in chondrocytes and chondrogenic differentiation of MSCs will result in the creation of cell-based therapies for the disease of cartilage degeneration, as well as manageable preconditioning methods for anatomically shaped MSC-based cartilage replacements.

We believe that the application of various modifications and mechanical stimuli of scaffolds can significantly advance tissue engineering by simulating the natural mechanical environment.
